# Techno-Economic Analysis of a Carbon Molecular Sieve-Based Xylene Isomer Purification Process

**DOI:** 10.1021/acs.iecr.4c02180

**Published:** 2024-10-11

**Authors:** Conrad J. Roos, Hammed A. Balogun, Ryan P. Lively

**Affiliations:** School of Chemical and Biomolecular Engineering, Georgia Institute of Technology, Atlanta, Georgia 30332, United States

## Abstract

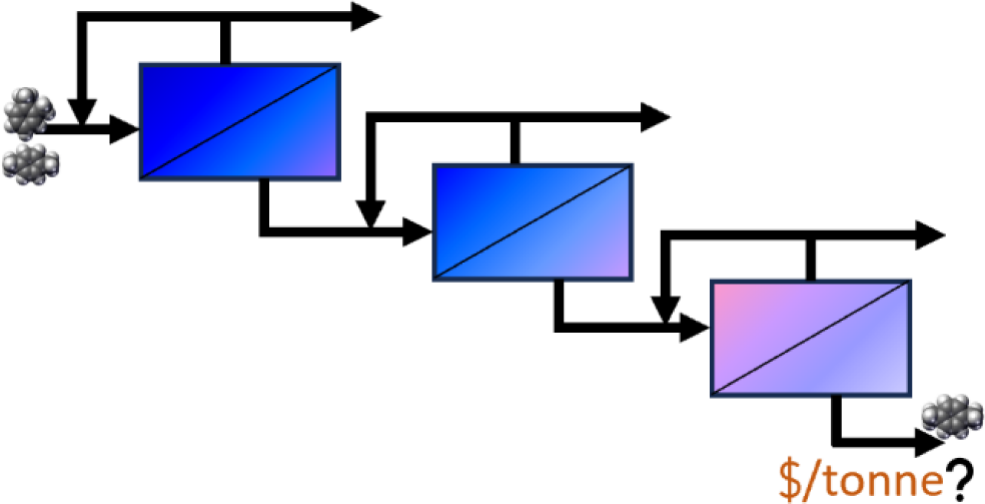

The separation and purification of xylene isomers is critical to the production of polyethylene terephthalate (PET). These separations are complex to operate and demand tremendous amounts of energy and thus are an opportunity for reducing industrial energy consumption. Membranes provide a low-energy footprint technology with simpler operation, and recently, membrane materials have been developed that are capable of separating xylene isomers. While these materials have demonstrated the ability to separate xylenes, they have yet to be commercially deployed. This work conducts a techno-economic analysis (TEA) to provide insight on the commercial attractiveness of a *p*-xylene selective carbon molecular sieve (CMS) membrane from both a cost and energy standpoint. This TEA was conducted through the pairing of a Maxwell–Stefan transport framework for rigid microporous materials with process modeling. Single-stage organic solvent reverse osmosis (OSRO) and pervaporation processes were used to evaluate the effects of recovery, selectivity, and diffusivity on the energy intensity and cost of the xylene separation. The final analysis used two systems, a single pervaporation stage followed by two OSRO stages, which was compared against a three-stage OSRO cascade. These were benchmarked against the commercial Parex process. We estimate that the membrane processes have the potential to enable impressive cost savings compared to the Parex process. While both systems outperformed the Parex process in terms of cost, the pervaporation/OSRO hybrid process was able to achieve the lowest cost of all due to its reduced membrane surface area compared to standalone OSRO. These findings demonstrate the potential of membrane systems in the field of difficult small molecule solvent separations.

## 1 Introduction

The separation of xylene isomers represents one of the most difficult separations in the chemical process industry.^1^ While the separation of these isomers through traditional distillation means is impractical as the similar physical properties would require an unfeasible number of trays, the value of pure xylene isomers necessitates high quality separation technology. *p-*Xylene is the most valuable of these isomers as it is a vital precursor in the fabrication of polyethylene terephthalate (PET), while *o-*xylene and *m*-xylene are largely used for the synthesis of phthalic anhydride and isophthalic acid respectively (to put into perspective the value of *p*-xylene, 80% of all xylene production is attributed to *p*-xylene).^2,3^

The current commercial separation of xylene isomers occurs through adsorption and crystallization, with the former accounting for roughly 75% of global xylene separations. The first commercial xylene purification was crystallization, which is possible due to the significant freezing point difference between *p*-xylene and the other isomers. Currently accounting for 25% of xylene purification worldwide, crystallization has been relegated to handling *p-*xylene-rich feedstocks.^2^ Adsorption-based separations currently dominate xylene purification due to its higher efficiency and lower energy consumption when compared to commercial crystallization processes. The main commercial systems such as UOP’s Parex, Toray’s Aromax, and IFP’s Eluxyl, utilize a simulated moving bed (SMB) process to produce *p*-xylene streams with purities greater than 99.7% and recoveries exceeding 97%.^4^

The SMB process is a simplification of the true moving bed (TMB) method of separation. In adsorption-based separation processes, purity is achieved due to differences in affinity between mobile phase components and the adsorbent. The component with lower affinity to the adsorbent in effect has a higher velocity through the bed than the higher affinity component. The SMB splits the adsorbent bed into zones, and instead of a moving bed as in TMB, a valve switches between the zones. Extended descriptions of this process can be found in the literature.^5,6^ On top of the complexity of operation, the SMB xylene purification processes require downstream distillation columns to separate purified xylene from the desorbent solvent. This is nonideal as distillation is often energy-intensive and raises the energy requirements and costs of xylene separations.^7^

Membranes, as a separation technology, offer the potential to reduce the cost and energy consumption of xylene isomer purification.^7^ The success of membrane technologies in water and gas separations points to their potential in addressing liquid hydrocarbon separations.^8,9^ They have already seen success in addressing some organic solvent nanofiltration applications (e.g., MAX-DEWAX, and processes enabled by Puramem and Duramem). In this work, we take the test case of *p*/*o*-xylene as a separation to investigate the potential of high-performance membranes to reduce the cost and energy demands of small molecule solvent separations. This difficult isomer separation provides a good test case and serves as a proof of concept for OSRO-based membrane processes. While no commercial membrane exists to separate *p/o*-xylene, there have been promising membranes developed on the lab scale.^10 −17^

One commonly used membrane material for this separation in vapor permeation or pervaporation modality are MFI-type zeolites.^12−14,17^ For instance, in the vapor permeation study conducted by Sakai and coworkers, *p-*xylene/*o-*xylene separation factors of ∼250 were reported at temperatures above 450 K.^13^ Xomeritakis et al. recorded a separation factor of 60–300 with the introduction of an additional solvent (*n*-hexane) or surfactant modification of their membranes for crack sealing in another vapor permeation study at 373–398 K.^12^ With the introduction of hierarchical layers, Liu et al. were able to achieve a high separation factor of 1228, the highest reported so far for MFI zeolite membranes.^17^ However, besides the need for expensive supports, these polycrystalline zeolite membranes often fail to maintain their idealized structures under ambient temperature and high xylene loading.^18^ Another interesting material for xylene separation is graphene oxide membranes with a separation factor of 2–4 reported for a liquid phase feed mixture of 50–90% *p-*xylene.^16^ Metal-organic framework membranes have also been implemented for xylene separation with a separation factor of 38.5.^15^ One other example is a hollow fiber carbon molecular sieve (CMS) membrane developed previously in our lab.^10^ This membrane benefits specifically from the demonstrated scalability of hollow fiber membranes paired with a highly selective CMS skin layer comparable to literature without further membrane modification to carry out separation at near ambient temperature.

A detailed description of the sorption-diffusion mechanism for xylene transport in the CMS membrane can be found in our previous work.^10,18,19^ Briefly, the CMS membrane properties chosen were based on prior experimental work using the pyrolysis of polymer of intrinsic microporosity (PIM-1) at 500 °C in a 4 vol % H_2_ atmosphere. In the OSRO modality, a *p-*xylene/*o-*xylene separation factor of ∼7 with a diffusive selectivity of ∼30 and similar sorption properties were reported for a feed mixture of 90 mol % *p-*xylene in the binary mixture.^18,19^ The permselectivity, mainly driven by diffusion, is a result of the ultramicropores within the CMS membranes.^18^

In this study, membranes operating in a pervaporation regime will serve as a comparison to the OSRO membranes. Pervaporation is a well-known membrane-based separation process for solvents and other liquids. Pervaporation has a liquid feed, but unlike OSRO, the permeate is a vapor under significantly reduced pressures. This creates a large driving force (relative to OSRO) as the downstream activity is effectively zero. The downside of this is that the vaporization of the permeate at the downstream face requires a significant amount of energy. Unlike OSRO separations, solvent pervaporation separations have been commercialized for many years.^20^

This paper seeks to explore the economic feasibility of xylene purification using advanced membrane systems. This work utilizes a Maxwell-Stefan transport framework designed for rigid microporous materials, nested within an Aspen Plus process model. Single-stage pervaporation and OSRO processes will be used to investigate the effects of membrane diffusivity, selectivity, and system recovery on permeate purity and energy consumption. Multistage processes (a three-stage pervaporation/OSRO hybrid and a three-stage OSRO cascade) are used to evaluate the economic potentials of membrane-based xylene separations. They are benchmarked against the state-of-the-art Parex process designed by UOP.^21^ Due to the limited information available on the energy consumption of commercial Parex processes, only a direct cost comparison can be made between Parex and the membrane processes. This work does not seek to investigate the effects of external mass transfer, membrane morphology, or hybrid nonmembrane processes, although we do recognize the immense potential for process improvements within these areas. Overall, this work seeks to identify critical parameters in separating xylene isomers using membranes and how they compare against current state-of-the-art technologies.

## 2 Methods

### 2.1 Process and Economic Modeling

Process modeling environments (PMEs) provide a straightforward tool for designing and testing industrial processes. The range of unit operations and versatile algorithms to drive system solutions provide a convenient environment for modeling these operations. While PMEs generally offer a robust selection of unit operations, they fall short of providing a wide range of membrane processes. To remedy this shortcoming, external membrane models have been developed that can run within PMEs.^22^

PMEs provide a powerful tool to those investigating processes; it is impractical for any single program to be capable of modeling the almost limitless combinations of unit operations, chemical properties, and models. To address this limitation, the CAPE-OPEN Interface Standard was conceived in 1997 to establish interoperability between the numerous modeling environments, property databases, and possible in-house models/databases. The CAPE-OPEN standard eliminates the need for an end user to create bespoke interfaces to achieve interoperability between their various tools. In the following work, a customized CAPE-OPEN interface allows for executing MATLAB scripts within the Aspen Plus PME.^23^

**Figure 1:**
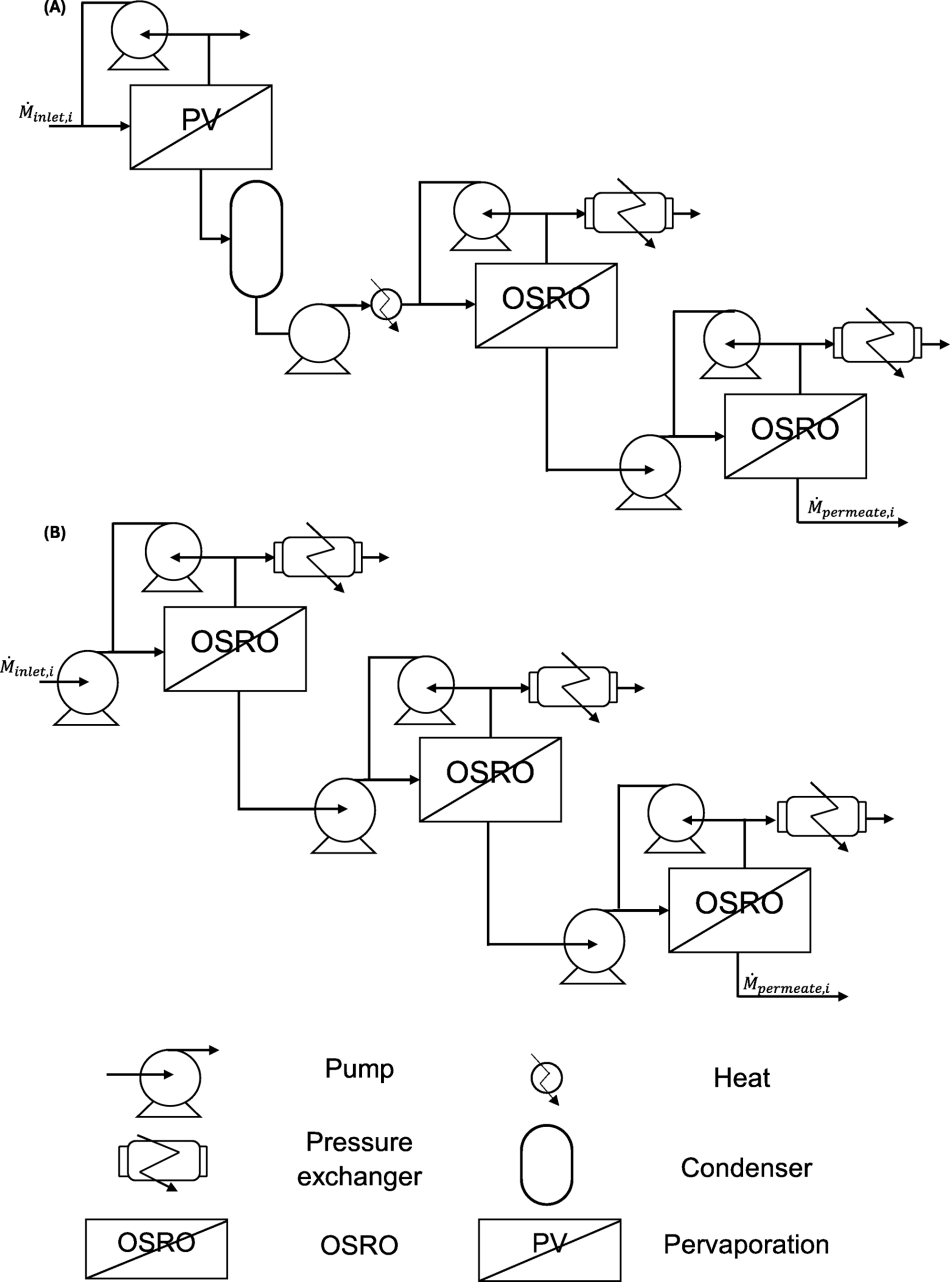
Complex membrane cascades for techno-economic analysis of xylene separation A) Pervaporation membrane followed by a two-stage OSRO cascade B) 3-stage OSRO cascade. Ṁ_inlet,i_ is the flow rate of component i fed to the separation process and Ṁ_permeate,i_ is the permeate flow rate of i from each membrane module (PV or OSRO).

Aspen Plus V10 was chosen as the PME for this work and was combined with Aspen Properties for *p*-xylene and *o*-xylene physical property data, with Peng–Robinson chosen as the equation of state. Aspen Plus currently lacks a robust membrane unit operation. Within our PME, this was addressed through the use of a MATLAB-based membrane unit, utilizing Maxwell-Stefan multicomponent transport equations for our chosen membrane. Aspen Plus provided the unit with feed parameters, and the custom unit operation provided the PME with composition and fluxes for both permeate and retentate. In this way, Aspen Plus, CAPE-OPEN, and MATLAB were used together to predict performance for single-stage OSRO, single-stage pervaporation, pervaporation/OSRO hybrid, and the 3-stage OSRO systems (Figure 1). For each membrane module, a maximum stage cut of 30% was implemented to minimize concentration polarization of the membrane. While the single-stage systems were used to understand the effects of recovery, membrane diffusivity, and membrane diffusion selectivity on process energy demand and cost, multistage processes were compared on a cost basis against the state-of-the-art Parex process. Sensitivity analyses are conducted to determine the impact of membrane surface area cost and *p*-xylene recovery on the process cost.

Beyond simulating the process parameters and final permeate purity, the economics of the system were also calculated. The majority of cost calculations were conducted using the Aspen Process Economic Analyzer (APEA). Due to the external nature of our membrane unit operation (run in MATLAB), APEA was unable to provide pricing for the membrane modules. For this analysis, the cost of membrane surface area was assumed as $50/m^2^. This analysis was conducted on a 10-year horizon with an assumed membrane lifespan of 5 years and negligible performance degradation due to the nature of the feed mixture. Future process costs were discounted to a present value (discount rate: 2%). Final production costs over the 10-year period were normalized to the mass of *p*-xylene produced over that 10-year period. The size of our modeled process plant was benchmarked at 72 kmol/h (∼57 kilotonnes per annum (kta) – based on an 85% plant yearly operational time) of inlet xylene mixture to allow for plant’s outlet flow rates to be within those reported for the Parex process.^21^ Detailed diagrams of both systems and their process parameters can be found in the Supporting InformationSections S1 – S2.

### 2.2 Maxwell–Stefan Transport Model

Solvent transport through rigid microporous and ultramicroporous materials occurs through a sorption-diffusion modality, which describes both pervaporation and OSRO transport. Unlike classical hydraulic filtration, transport by the sorption-diffusion mechanism does not occur due to a pressure gradient within pores of the membrane, but is instead driven by a pressure-induced activity gradient across the membrane.^24^ The transition from a pressure gradient to a pressure-induced activity gradient occurs as continuum flow breaks down. The breakdown in continuum flow occurs as the pore size shrinks to around 2–3x the smallest molecular cross-section of the guest molecule.^25^ A benefit of the sorption-diffusion model is that it allows for the permeability to be described as a product of diffusive and sorption contributions, displayed in eq 1,
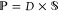
1where  is permeability, *D* is the transport (or Fickian) diffusivity, and  is the sorption coefficient. While Fickian diffusivities are relatively easy to measure and thus are widely used in mass transport studies, their shortcomings in multicomponent systems are well documented. These shortcomings have created enduring interest in the application of the Maxwell-Stefan (MS) transport framework. The MS transport model provides a more accurate picture of multicomponent transport by accounting for component interactions. The benefits of the MS framework have driven refinements in how the framework can describe transport in microporous membranes, along with expanded applications in these materials.

Krishna and Baur developed a MS equation for transport through microporous materials.^26^
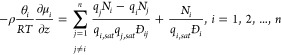
2

where *z* represents the membrane’s depth, the component fractional occupancies are defined as
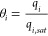
3with *q*_*i*_ being the molar loading of species *i* and the saturation loading being *q*_*i,sat*_. *Đ_i_*, known as the single component MS diffusivities accounts for the effect of the framework on the penetrating species. These diffusivities can be calculated from Fickian diffusivities through the application of a thermodynamic correction factor, shown below.^19^
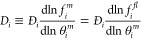
4with  being the fugacity of component *i* in the fluid phase and  as the component fractional occupancy in the membrane. eq 4 successfully removes the effect of sorption from the transport diffusivity, and in the case of simple Langmuir sorption can be simplified to
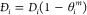
5where
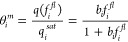
6with  being the saturation loading of the penetrant within the membrane, and *b*_*i*_ is the Langmuir affinity constant. In this work, we describe the sorption characteristics of our membrane using the Langmuir isotherm, as the Langmuir isotherm has been shown to accurately describe the membrane of interest in prior work.^18^ If the material of interest cannot be described using a Langmuir isotherm, one must start at eq 4 and rederive eq 5 using the applicable isotherm.

The *Đ*_ij_ terms shown in eq 2 are commonly referred to as exchange coefficients. These coefficients represent the coupling or “drag” between two species. The physical manifestation of this coupling effect is the slowing of faster penetrants and a faster transport of the slower species. The larger the value of *Đ*_ij_ the less pronounced this coupling is. The Vignes correlation, a logarithmic interpolation, is commonly used to estimate this coupling and is shown below:^27^
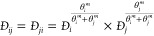
7

To provide a more workable form of the MS framework, eq 2 can be reformatted as a set of matrices, as shown in eq 8.^19,26^
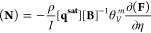
8

Where ρ is the density of the microporous material,  is the membrane thickness, η is a dimensionless position along the length of the membrane, **F** is a dimensionless fugacity where  with *b*_*i*_ being the Langmuir affinity constant, **N** is a matrix of the molar fluxes, and [**q**_**sat**_] is a diagonal matrix of the saturation loadings. The components of the diffusion matrix, [**B**], are
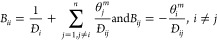
9

The diffusion and sorption values of *p-* and *o-*xylene in the CMS membrane are shown in Table 1. The chosen values were anchored around the values for a carbon molecular sieve membrane previously reported by our group.^19,28^ This work did not adjust the sorption values for the membrane but did investigate the effects of changing diffusivity. In the cases where the membrane’s diffusion properties were changed, the membrane’s *p*-xylene diffusivity and diffusion selectivity, shown in eq 10, were altered by Aspen Plus using the CAPE-OPEN interface. It was decided to alter the membrane’s diffusion properties as it is relatively difficult, especially in the case of xylene isomers, to impart significant sorption selectivity onto the membrane.
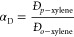
10where α_D_ is the diffusion selectivity of the membrane for *p*-xylene over *o*-xylene.

**Table 1:** Maxwell-Stefan Diffusivities and Langmuir Model Parameters for Para- and Ortho-Xylene in a Carbon Molecular Sieve Membrane.^19^^a^

	Value^b^
*p*-Xylene Langmuir affinity constant, *b*_*p-xylene*_ (kPa^–1^)	3.8
*o*-Xylene Langmuir affinity constant, *b*_*o-xylene*_ (kPa^–1^)	3.2
*p*-Xylene saturation loading, (mmol/g)	1.3
*o*-Xylene saturation loading, (mmol/g)	1.3
*p*-Xylene diffusivity, *Đ*_*p-xylene*_ (cm^2^/s)	2.7 × 10^–10^ (10^–12^ – 10^–8^)
*o*-Xylene diffusivity, *Đ*_*o-xylene*_ (cm^2^/s)	8.7 × 10^–12^
Diffusion selectivity, α_D_	30 (10–90)

a Adapted in part with permission from Ma et al. Ind. Eng. Chem. Res. 2020, 59, 12, 5412–5423. Copyright © 2019 American Chemical Society

b The value in parentheses shows the range of parameters that are varied in sensitivity analysis.

The CAPE-OPEN interface also enabled the investigation of changing *p*-xylene recovery, shown in eq 11, on the separation performance. The target recovery was passed into the MATLAB script through the CAPE-OPEN interface. After the solvent fluxes were calculated, the recovery was used to calculate the surface area necessary to achieve that *p*-xylene recovery. These surface areas were transferred to Aspen Plus.
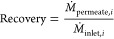
11where *Ṁ*_*i*_ is the flow rate (molar or mass) of the desired component in either the inlet or permeate.

Modeling of permeation through the rigid microporous membrane using the MS transport framework used the following assumptions:

Sorption-diffusion transport
No external mass transfer limitations
Langmuir sorption behavior^19,28^
Isothermal membrane layer
*Đ_im_* is independent of loading

And utilized the following boundary conditions:

Upstream face

z = 0; η = 0;

Downstream face

z = ; η = 1; 

A flowchart has been provided in Supporting Information outlining how the MS transport framework was utilized to solve for fluxes and downstream compositions (see Supporting Information Section S3). Ma et al. and Krishna and Baur provide a more detailed accounting of how the MS framework utilized in this work was developed.^19,26^

## 3 Results and Discussion

### 3.1 The Role of Recovery within Membrane Separations

**Figure 2:**
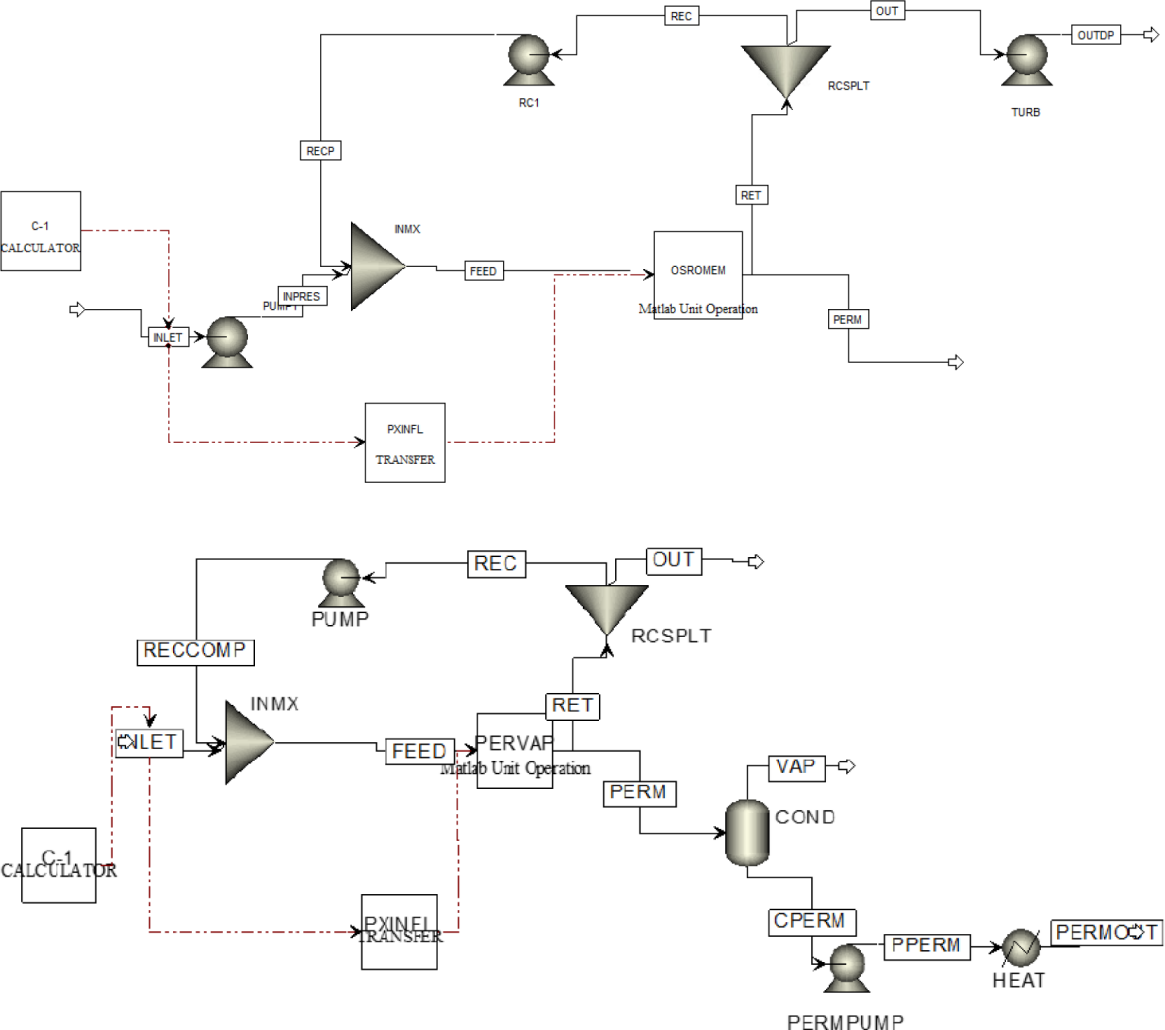
Process flow diagrams for single-stage OSRO (top) and pervaporation (bottom) processes.

The recovery of the desired product is a critical parameter of interest for any separation system as it directly relates to the productivity of the unit operation. While higher recoveries result in greater yields (i.e., *p*-xylene permeated through the membrane), there are trade-offs within membrane systems that can make higher recoveries less desirable. Here, we specifically focus on how recovery affects the permeate purity and energy consumption for membrane systems. The ASPEN process flow diagrams for the single-stage OSRO and pervaporation membranes are shown in Figure 2. In both cases, recycle streams are considered to keep stage cuts low and recoveries high.

**Figure 3:**
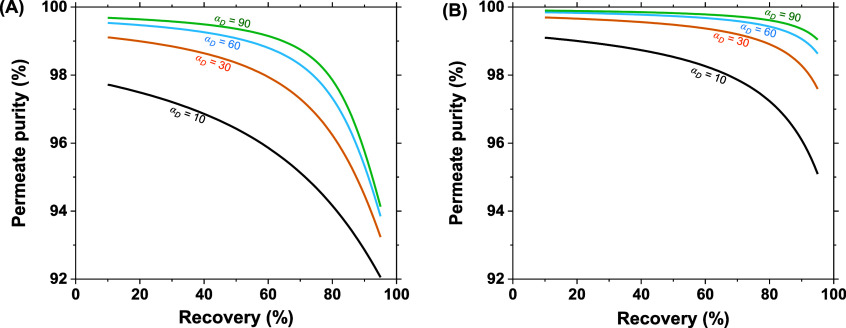
Permeate purity as a function of recovery for single stage A) OSRO and B) pervaporation membranes with varied diffusion selectivities (α_D_). Black denotes α_D_ of 10, orange 30, blue 60, and green 90. Experimental diffusion selectivities for these membranes are around 30.^10,28^ The inlet *p*-xylene molar composition is 90%, and outside of recoveries greater than 93%, stage cuts were under 30%. Transmembrane pressure is 100 bar for OSRO and a vacuum pressure of 0.005 bar for pervaporation systems.

In Figure 3, the effect of recovery on the permeate purity of single-stage OSRO and pervaporation membranes with varied selectivities is shown. The most apparent difference between the OSRO and pervaporation systems is their permeate purities. Figure 3A, showing the permeate purities for the OSRO process, demonstrates lower *p*-xylene compositions across all selectivities and recoveries when compared to those provided by the pervaporation process in Figure 3B. This difference is directly related to the driving forces associated with each process. The pervaporation system has a relatively deep downstream vacuum (∼0.5 kPa), resulting in a downstream membrane activity of nearly zero for the permeating components. This resultant driving force surpasses that of the liquid phase OSRO process, which ultimately enables higher purities for pervaporation. The effect of membrane selectivity is decidedly more simple. As the selectivity of the membrane increases, the purity of the permeate increases regardless of separation modality or recovery.

Across both pervaporation and OSRO, we see declines in *p*-xylene composition in the permeate as the system’s recovery increases. This decrease results from the combination of changing recovery and a constant recycle stream. As the recovery is raised, the retentate’s *p*-xylene composition decreases. A portion of the retentate is recycled in this “feed and bleed” system, causing the *p*-xylene composition in the feed to decrease with increasing *p*-xylene recovery. As described by the sorption-diffusion model, the driving forces for transport are the component’s chemical potential gradients which are directly influenced by their composition. Thus, a lower *p*-xylene feed composition yields a lower driving force, which ultimately results in a decreased *p*-xylene composition in the permeate. This trade-off between recovery and permeate purity will necessitate a balancing act for any potential membrane-based xylene separation (Section S4).

**Figure 4:**
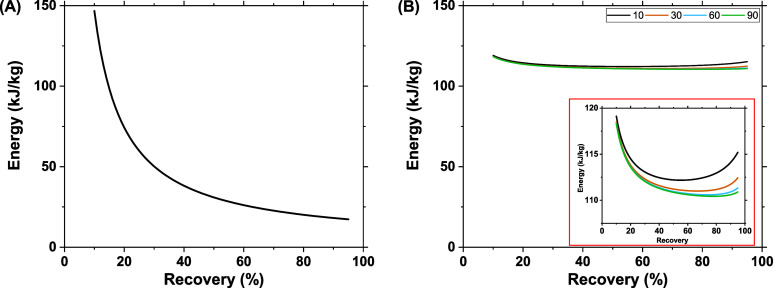
Energy cost per kg of *p*-xylene product as a function of recovery for single stage A) OSRO and B) pervaporation membranes with varied diffusion selectivities (noted in legend). The inlet *p*-xylene molar composition is 90%, and all stage cuts are <30% at recoveries <93%. Transmembrane pressure is 100 bar for OSRO and a vacuum pressure of 0.005 bar for pervaporation systems. Inset in (B) highlights the trend in pervaporation system energy cost across different selectivities.

While the effects of recovery on permeate purity follow the same general trend for pervaporation and OSRO, it is quickly apparent that this is not the case for the energy consumption of the two processes. Figure 4 illustrates the energy demands of the two membrane systems, with multiple selectivities, as the system recovery changes. For the OSRO system, displayed in Figure 4A, we see that the energy consumption does not noticeably change with membrane selectivity. While there are small changes in the permeate flow rates as a result of selectivity changes, as evidenced in Figure 4A, these only slightly change the retentate flow rate. While changes in retentate flow rate will change the energy consumption, any repressurization energy penalty from the recycle is largely offset by the pressure recovery unit (energy/pressure exchanger) on the outlet. However, the energy gained or lost from either of these unit operations pales in comparison to the energy required to pressurize the inlet, which is constant across all cases. This large energy demand on the initial pressurization is the primary reason only minor differences are observed across OSRO selectivities.

The pervaporation system demonstrates fairly significant differences in energy consumption as the membrane’s selectivity changes. These are due to two key differences from the OSRO process: lack of a pressurized feed and a vapor permeate. The energy required to pressurize the inlet stream for the OSRO process far surpassed the selectivity-induced energy variations, minimizing their apparent influence on the total process. The large variations in energy consumption as a function of membrane selectivity are largely driven by the pervaporation process’s downstream condenser. Like the inlet pump in the OSRO case, the condenser accounts for the largest energy demand in the pervaporation case. As selectivity drops so does the downstream *p*-xylene composition, yet the constant recovery keeps the *p*-xylene flow rate the same. This results in larger permeate flow rates as the selectivity drops, which requires larger amounts of energy to condense. In comparison, OSRO outperforms pervaporation for *p-*xylene separation except at extremely low *p-*xylene recovery, due to high pumping power for inlet feed with low *p-*xylene recovered (Figure S5). Figures S6 and S7 show the distribution of the process energy requirement by each equipment. In OSRO, the system energy is mainly dominated by the feed pressurization pump, while for pervaporation, the heating energy for the membrane module is greatly offset by the recovered energy of the permeate condenser.

### 3.2 Understanding the Role of Differing Selectivities

To evaluate the economic feasibility of a membrane-driven xylene isomer separation, we must understand how major membrane characteristics affect the process cost. Thus, this analysis focuses primarily on cost sensitivity with respect to the membrane and not through process optimization. The first area we seek to understand is the relationship between the membrane’s diffusion selectivity and the cost.

**Figure 5:**
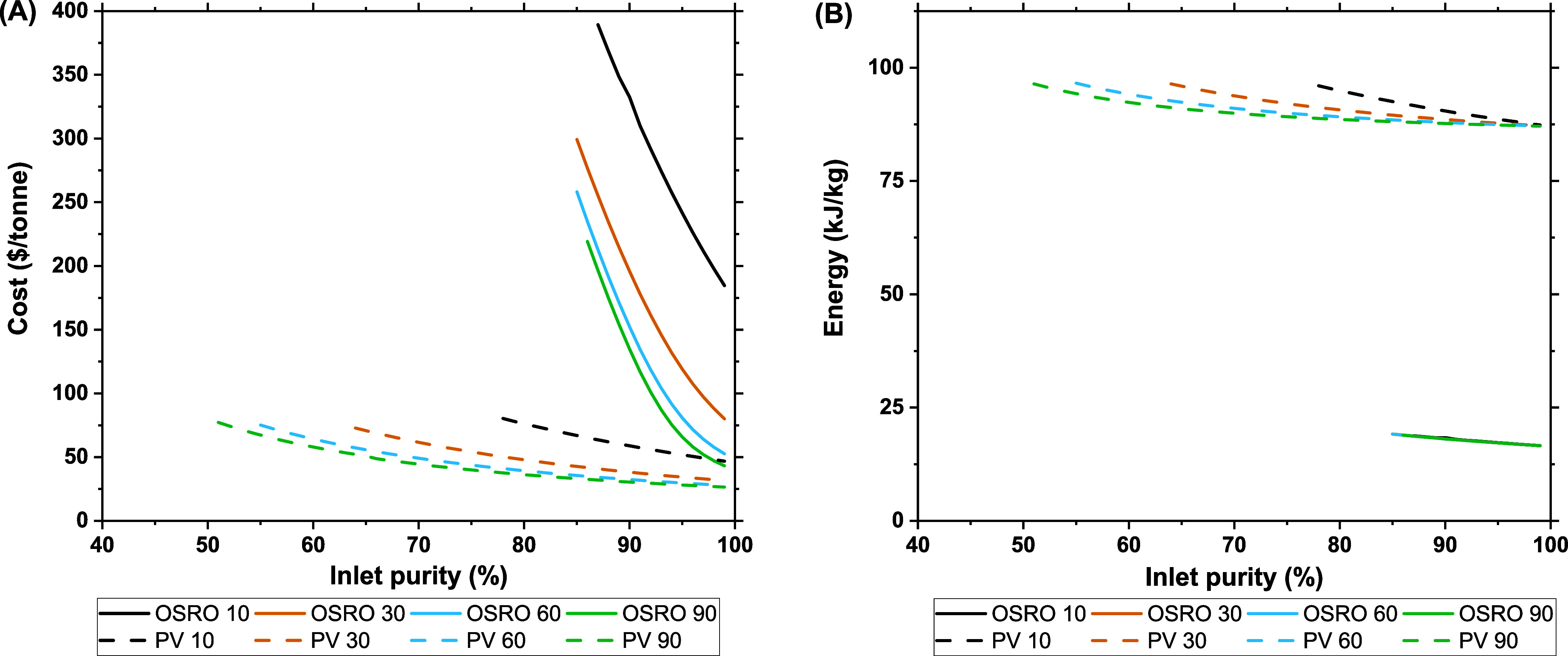
Cost per tonne for single-stage OSRO and pervaporation processes B) Energy consumption (kJ/kg) for single-stage OSRO and pervaporation processes (experimental α_D_ = 30). Cost and energy consumption are normalized per unit of *p*-xylene product. All membranes had an o-xylene diffusivity of 8.74 × 10^–12^ cm^2^/s; all displayed data have permeate *p*-xylene compositions >90%, and *p*-xylene recovery is set at 90%. Transmembrane pressure is 100 bar for OSRO and a vacuum pressure of 0.005 bar for pervaporation systems with a feed temperature of 323 K.

Figure 5A visualizes how the cost per tonne of *p*-xylene product changes across varied inlet composition for certain diffusion selectivities. The largest takeaway from Figure 5A is the price difference between the OSRO and pervaporation systems. This price difference stems directly from the surface area necessary to reach the system’s target recovery of 90%. The higher driving force found in the pervaporation process produces higher fluxes relative to the all-liquid OSRO process. These higher fluxes result in lower surfaces areas required to achieve the necessary recoveries. Within each system, we observe a drop in the cost per tonne as we go to higher selectivities, stemming from a decrease in the necessary membrane surface area to maintain a *p*-xylene recovery of 90%.

Figure 5B shows the variation in the energy consumption of these processes change with inlet composition. We estimate a substantial difference between the pervaporation and OSRO processes. This difference largely stems from the two processes permeate. In the case of OSRO, the permeate is a liquid, while in pervaporation it is a vapor. The condensation of the permeate vapor, which doubles as vacuum source in this simulation, requires a substantial amount of energy and is responsible for the difference in energy consumption between OSRO and pervaporation. As the selectivity of the pervaporation membrane increases, the energy requirements decrease. This is a due to the reduced permeate flow rate from higher selectivity membranes. The more selective a membrane, the less *o*-xylene present in the permeate. As mentioned in the previous paragraph, the condensation step of the pervaporation processes dominates its energy consumption. Thus, it follows that as the total flow rate of permeate decreases so too does the energy necessary to condense that stream, resulting in lower energy consumption for more selective membranes.

### 3.3 Understanding the Role of Differing Diffusivities

The membrane’s *p*-xylene diffusivity plays a significant role in determining the required membrane surface area as it is directly linked to the membrane’s throughput. The direct relationship between membrane surface area and *p*-xylene diffusivity results from dictating the system’s *p*-xylene recovery. As mentioned earlier, this turns the required membrane surface area into a function of *p*-xylene flux through the membrane, which is directly affected by the membrane’s *p*-xylene diffusivity (see Figure S8).

**Figure 6:**
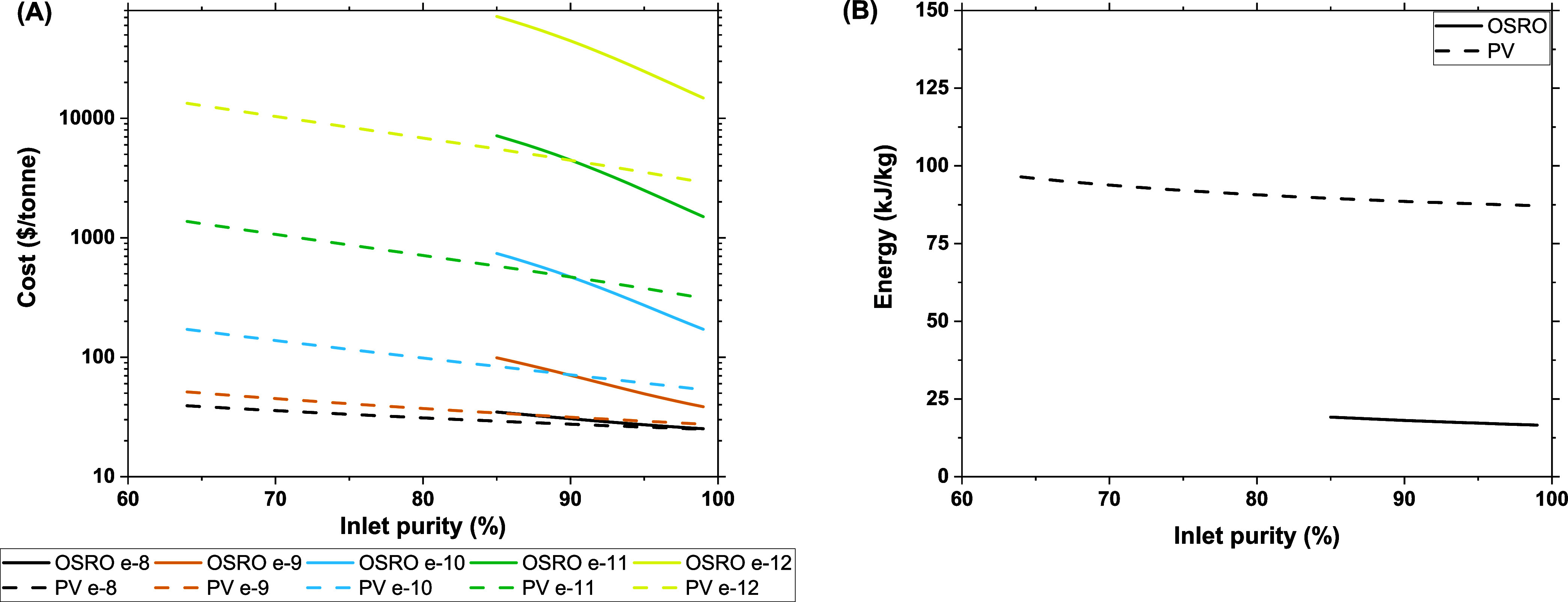
Varied *p*-xylene diffusivities and its effect on A) cost per tonne and B) energy consumption (kJ/kg) for two single-stage OSRO and pervaporation systems (experimental D_p-xy_ of 10^–10^ cm^2^/s). Cost and energy consumption are normalized per unit of *p*-xylene product. The diffusion selectivity of all membranes is 30; recovery is set at 90%, and all displayed data have *p*-xylene permeate purities exceeding 90%. Transmembrane pressure is 100 bar for OSRO and a vacuum pressure of 0.005 bar for pervaporation systems. A *p*-xylene diffusivity of 10^–8^ cm^2^/s corresponds to a hydraulic permeance of 4.53 Lm^2–^h^–1^bar^–1^ for the OSRO operating conditions, and a true permeance of 7.14 cc(STP)cm^–2^s^–1^cmHg^–1^ for the pervaporation conditions (see Section S10).

Figure 6A compares the cost per tonne of *p-*xylene product for both the pervaporation and OSRO example systems across multiple *p*-xylene diffusivities, with the diffusion selectivity held constant. The most noticeable result on this plot is that higher diffusivities lead to cost reductions across both systems. As the *p*-xylene diffusivity increases, the flux of *p*-xylene through the membrane increases accordingly. This results in less surface area required to meet the target recovery of 90%. While this relationship is not particularly profound, the effects of reducing membrane surface on costs are. The data in Figure 6A suggests the relationship, but the information presented here demonstrates that membrane material costs largely determine the total process costs. While this does stem directly from the assumed cost of the membrane material ($50/m^2^), the order of magnitude cost difference between diffusivities suggests that costs associated with the membrane material will largely dictate process costs. Only in processes with high diffusivity membranes (approaching 1 × 10^–9^ cm^2^/s) do the further increases in *p*-xylene diffusivity have a reduced impact on process costs.

Comparing the two systems yields the same conclusion as Figure 6A. The pervaporation system consistently outperforms the OSRO system on a cost basis. The lower driving force inherent in the OSRO separation requires greater surface areas to provide the same throughput as the pervaporation unit.

As shown in Figure 6B, differences in diffusivity within the same system do not meaningfully change the energy consumption of the membrane process. As discussed in the previous section, change in energy consumption results directly from changing flow rates in the process streams. Changing the diffusivity of a membrane will directly affect the flux and, thus, the flow rates exiting the membrane. But specifying the *p*-xylene recovery breaks this relationship. The target recovery sets the *p*-xylene flow rate in the permeate. Any changes in *p*-xylene diffusivity will only change the amount of membrane surface area required to produce that flow rate. Therefore, as long as recovery is held constant, the membrane’s *p*-xylene diffusivity will not affect the energy demand of the process. In addition, pervaporation demands greater amounts of energy than a comparable OSRO system. This, once again, is attributable to the vapor permeate of the pervaporation process which necessitates a significant energy penalty for condensation.

### 3.4 Cost Analysis of the Single-Stage Membrane Processes

Understanding the cost distribution of each of the components in OSRO/pervaporation systems could help provide insight into critical units and opportunities for process improvement. To better understand the cost distribution, three different compositions of the process cost are considered: the capital cost, the operating cost (both capital and operating costs estimated by APEA for all other units except the membrane modules), and the membrane module cost.

**Figure 7:**
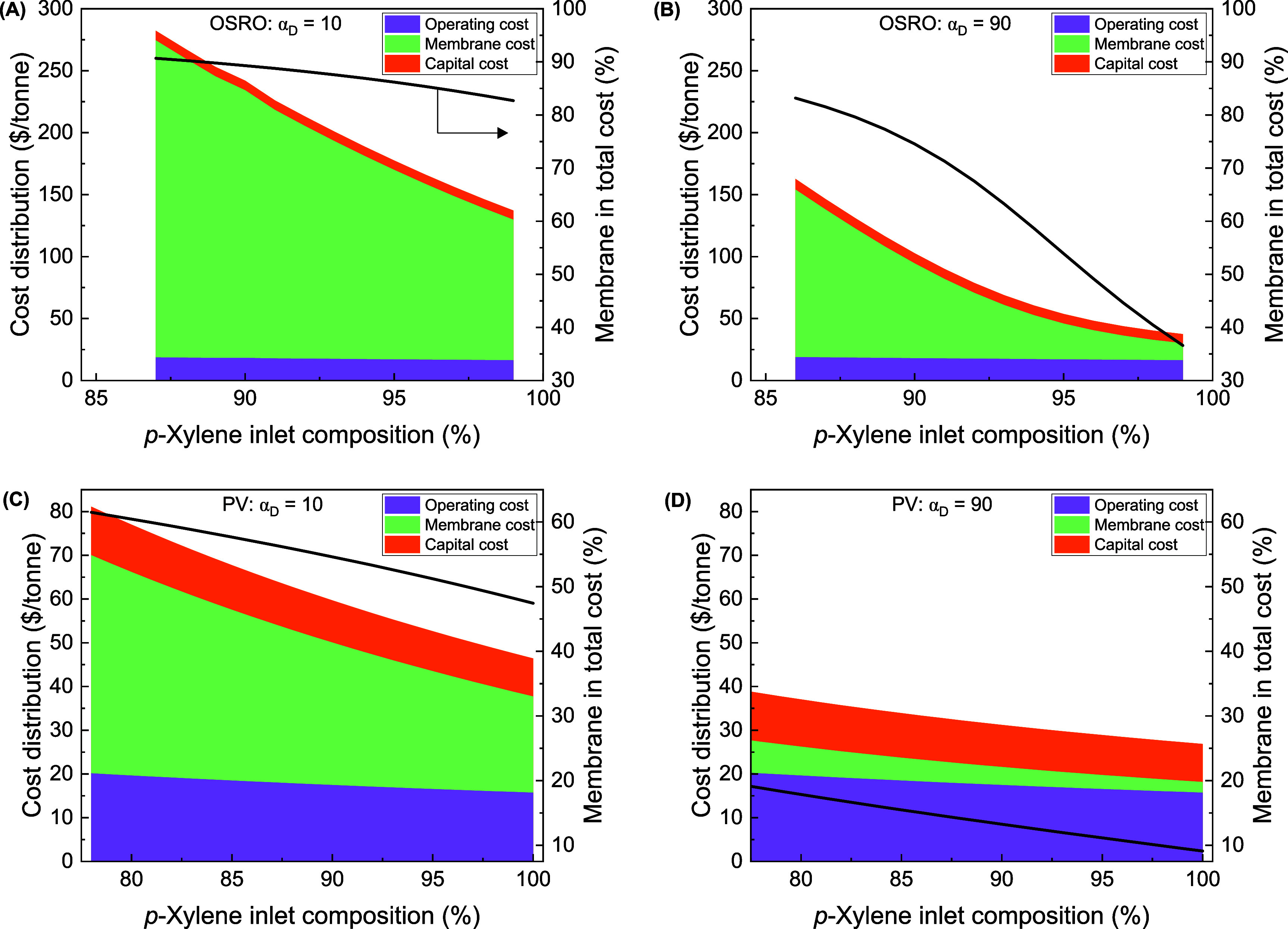
Analysis of cost components of single-stage OSRO (A and B) and pervaporation (C and D) processes for xylene separation at the high and low diffusion selectivities considered in this study. Cost components are shown in a shaded area, while the percentage contribution of the membrane modules to total cost is represented with a black line (on the right). The operating and capital costs were obtained from the Aspen Process Economic Analyzer. All membranes had an *o*-xylene diffusivity of 8.74 × 10^–2^ cm^2^/s; all displayed data have permeate *p*-xylene compositions >90%, and *p*-xylene recovery is set at 90%. Transmembrane pressure is 100 bar for OSRO and vacuum pressure of 0.005 bar for pervaporation systems with a feed temperature of 323 K.

Figures 7 and S9 show the contribution of annualized capital cost, membrane, and operating cost for the pervaporation and OSRO example systems at varying diffusion selectivities with constant *p*-xylene diffusivity. The most noticeable result on these plots is that higher *p*-xylene composition of the inlet feed mixture leads to drastic membrane cost reduction with negligible change in the other two cost components across both systems. As the diffusivity selectivity increases and with a target recovery of 90%, the influence of selectivity and inlet composition is mostly reported in the membrane cost contribution. In the same trend, the proportion of total cost accounted for by the membrane unit decreases with increasing selectivity. However, surprisingly for the OSRO process, at lower *p*-xylene feed composition (85%), the membrane contribution drops by just 7% of the total contribution, whereas for a nearly pure *p*-xylene feed (99%), the membrane accounts of the total separation cost decreases significantly from 83% to 36.5%.

Comparing the cost distribution for the separation of *p*-xylene concentrated feed (>96%), the membrane module remains the most determining factor in the OSRO process cost. This might be due to the low-energy nature of this separation, the lower flux, or the less complex equipment (pressure exchangers) needed with the membrane unit to achieve the separation. However, for pervaporation systems, the membrane module costs become appreciably lower fractions of the process costs compared to other units, such as the condenser and heat exchangers at high membrane diffusion selectivity and higher *p*-xylene composition of feed mixture. In addition, the trend of the membrane contribution becomes steeper with increasing diffusion selectivity for OSRO with no noticeable change for pervaporation systems due to the difference in driving force for the two membrane operations.

The sensitivity analysis of the process cost to change in membrane lifetime is another dimension to understanding the influence of the membrane units on the separation process cost. As discussed earlier, the OSRO process costs are more sensitive to changes in membrane lifetime as compared to pervaporation systems (see Figure S10). With the assumption of negligible membrane degradation during its lifetime, replacing the membrane modules every year would lead to an increase of 102% and 266% in pervaporation and OSRO process costs, respectively. This observation implies that OSRO membranes must be designed to ensure less frequent replacement, as this might drastically impact the economics of the separation process. The lesser sensitivity of the pervaporation membrane unit suggests that the process presents better economic competitiveness to the Parex process even with a high membrane replacement rate.

### 3.5 State-of-the-Art CMS Membranes Vs Parex

In this section a state-of-the-art CMS membrane is compared against the commercial Parex process^21,29^ to purify xylene isomers. The adsorption-based Parex process represents the state-of-the-art in commercial xylene separations and provides excellent xylene purities and recoveries.^29^ To provide a better comparison, the single-stage processes used in the preceding sections are replaced by multistage cascades. These multistage cascades provide greater *p*-xylene recoveries and purities in the permeate, boosting their commercial appeal.

**Figure 8:**
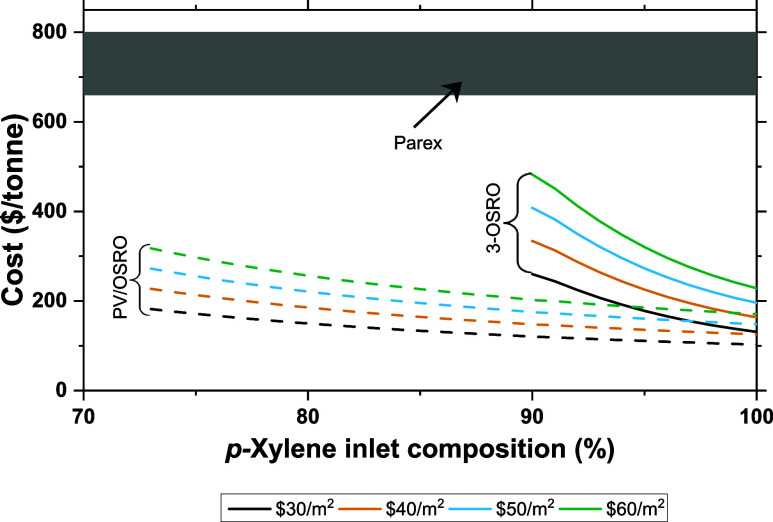
Cost per tonne of *p*-xylene product for pervaporation/two-stage OSRO hybrid (PV/OSRO) and a three-stage OSRO cascade (3-OSRO). Number in the legend denotes the cost per sq. meter ($/m^2^) of membrane for each system. The shaded band corresponds to the range of Parex costs (from Bhattacharya (2017)),^29^ all permeate compositions are >99 mol % *p*-xylene, and *p*-xylene recovery is 90%. Transmembrane pressure is 100 bar for OSRO and a vacuum pressure of 0.005 bar for pervaporation systems.

Both membrane systems consist of three stages. One system is entirely OSRO modules (Figure 1, bottom), while the other consists of a pervaporation stage followed by two downstream OSRO modules arranged in a cascade (Figure 1, top). This hybrid pervaporation/OSRO module design was chosen to avoid complicated process design with minimal unit operations to achieve comparable separation performance. However, more optimal designs than the ones studied here likely exist. The process costs for these two systems are plotted as a function of inlet *p*-xylene composition in Figure 8; only data with *p*-xylene permeate purities exceeding 99% are displayed. The different lines correspond to different assumptions of membrane cost ranging between 30–60 $/m^2^. The most apparent difference between the two multistage processes is that the pervaporation/OSRO system provides high-purity permeate (>99% *p*-xylene) at significantly lower *p*-xylene inlet compositions (for *p-*xylene permeate purity from the third membrane module across varying process inlet composition, see Figure S11). This is due to the greater *p*-xylene driving force in the pervaporation module compared to the all-liquid OSRO modules.

The greater driving force present in the pervaporation module is also responsible for the lower cost associated with the PV/OSRO hybrid. As discussed earlier, the larger driving force across the pervaporation membrane results in greater flux. The larger fluxes require less surface to achieve the recovery targets than the slower OSRO modules, resulting in lower costs associated with the pervaporation membrane.

Importantly, both systems are competitive against the Parex process from a cost standpoint. This competitiveness holds across a range of assumed membrane costs. There are clear limitations in the operating conditions of the multistage systems. The three-stage OSRO cascade is quite limited in terms of operating conditions as it can only provide high-purity permeate at *p*-xylene inlet compositions exceeding 90%. The PV/OSRO hybrid performs better but is limited to inlet compositions exceeding 73%. These limitations result from decreased driving forces at lower inlet compositions. These reduced *p*-xylene driving forces cannot produce high-purity (>99%) *p*-xylene permeates. While the two systems are highly competitive within these operating parameters, it places clear restrictions on their application, limiting them to relatively high-purity inlet streams.

**Figure 9:**
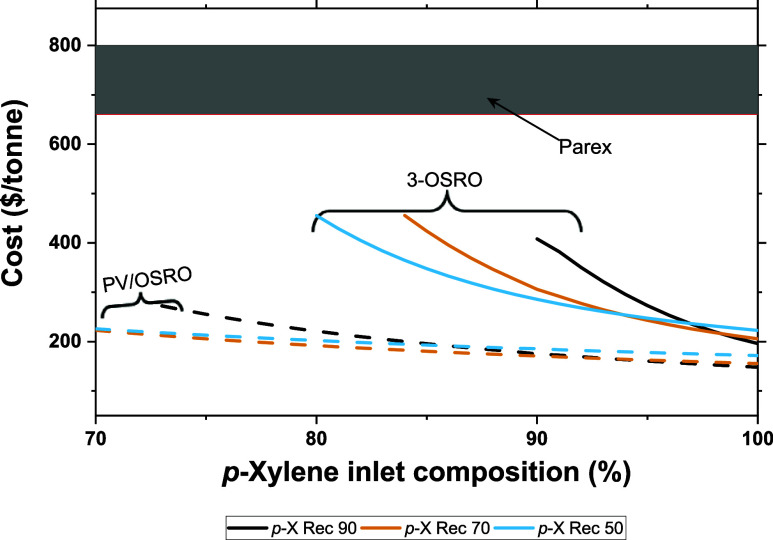
Cost per tonne of *p*-xylene product for pervaporation/two-stage OSRO hybrid (PV/OSRO) and a three-stage OSRO cascade (3-OSRO) across varied inlet composition. The number in the legend denotes the *p*-xylene recovery of the first module in each cascade; the succeeding modules have recoveries of 90%. The shaded band corresponds to the range of Parex costs (from Bhattacharya (2017));^29^ all permeate compositions are >99 mol % *p*-xylene, and the membrane cost is 50 $/m^2^. Transmembrane pressure is 100 bar for OSRO and a vacuum pressure of 0.005 bar for pervaporation systems.

One method of achieving higher *p*-xylene permeate compositions at lower inlet compositions is changing the system recovery. As described earlier in Section 3.1, lower recoveries provide higher *p*-xylene permeate compositions for the same conditions. Figure 9 shows the cost per tonne of *p*-xylene product across three recoveries for both the three-stage OSRO and PV/OSRO hybrid systems. In this analysis, only the recovery of the first membrane module was adjusted; the final two modules still operated at 90% recovery. This shows that by moving to a lower recovery for just the initial module, both systems could produce >99% *p*-xylene permeates at significantly lower inlet compositions (see Figure S11). This reduction in *p*-xylene greatly extends the operating range of the two systems without significant changes in cost.

While lowering the system’s recovery allows for higher purities at lower inlet compositions, this approach has clear downsides. Recovery measures the total amount of a compound extracted in a system compared to the amount of that compound fed into the process. Therefore, high recoveries are desired to prevent wasting valuable products. Expecting these membrane systems to operate as stand-alone processes would, therefore, preclude the possibility of using these membrane systems at lower recoveries. Running these membranes in a hybrid format is one method that could address this. The lower recovery membrane systems can provide a high-purity *p*-xylene permeate at dilute inlet concentrations for a lower cost than the Parex process. The dilute retentate exiting these membranes would then be fed into a Parex (or other adsorption-based processes) capable of separating the isomeric mixture. This hybridization would lower the effective cost of the total xylene purification process.

## 4 Conclusions

Maxwell-Stefan transport frameworks were incorporated into a PME (Aspen Plus) to evaluate the performance of both pervaporation and OSRO membranes in the separation of *para-* and *ortho-*xylene. While the membrane’s diffusion selectivity slightly influenced the process costs, the membrane’s *p*-xylene diffusivity had a more pronounced impact. This is because the membrane’s *p*-xylene diffusivity directly determines the membrane flux and, therefore, the membrane surface area required to meet a target recovery. The cost associated with the membrane material far exceeds all other operating or capital costs, so increasing solvent flux is critical in reducing the cost of the separation. This is the primary reason why pervaporation systems performed at lower costs than OSRO systems. Their higher driving forces necessitated less surface area to achieve the same recovery as an OSRO system.

Single-stage processes are useful in evaluating how process parameters and membrane characteristics influence the process’s cost and energy consumption but cannot produce permeate purities high enough for commercial interest. Two multistage processes were used to gauge the potential of membrane separations in xylene separations and were benchmarked against the adsorption-based Parex process. The PV/OSRO hybrid and three-stage OSRO cascade demonstrated excellent performance compared to the Parex process. The primary drawback of these two systems was their inability to provide high-purity permeate at dilute inlet conditions. This was countered by lowering the *p*-xylene recovery of just the first module in the system, greatly expanding the operating range of the process at the expense of total *p*-xylene throughput. While lower recoveries are unattractive for a stand-alone membrane system, a hybrid system composing a membrane cascade and a downstream Parex process could effectively lower the cost of *p*-xylene purification.

While this work provides useful insights into the effects of membrane characteristics on process costs and energy demands, some key limitations exist. The modeling work focuses almost entirely on membrane characteristics and not the process parameters. While the scope of this work focuses on those membrane characteristics, process optimization would be necessary if one wished to investigate the commercial potential further. Perhaps the main limitation of this work is omitting the effects of stage cut from the calculation. It is well-known that stage cut and concentration polarization substantially influence membrane performance. To avoid these complications, the stage cut was held under 30% for all runs. Further work could look to model the effects of stage cut to provide a more robust model capable of pushing into lower recycle ratios. Another key limitation of our work is the current unknown status of the lifetime of CMS membranes in practice. Our analysis suggests that this is a key factor in the overall costs, especially for the OSRO systems, and experimental data in this area is sorely needed.

## Supporting Information Available

Section S1. Three-stage OSRO cascade. Section S2. Three-stage pervaporation/OSRO hybrid cascade. Section S3. Maxwell-Stefan transport flowchart for xylene separation. Section S4. Permeate purity for 1-stage OSRO/pervaporation xylene separation process. Section S5. Normalized energy requirement for 1-stage OSRO/pervaporation xylene separation process. Section S6. Equipment energy demand for 1-stage OSRO/pervaporation xylene separation process. Section S7. Membrane module area for 1-stage OSRO/pervaporation xylene separation process. Section S8. Cost distribution and sensitivity analysis for 1-stage OSRO/pervaporation separation process. Section S9. Permeate purity from three-stage membrane cascade systems. Section S10. Permeance estimation(PDF)

## References

[ref1] Sholl D. S.; Lively R. P. Seven Chemical Separations to Change the World. Nat 2016, 532 (7600), 435–437. 10.1038/532435a. 27121824

[ref2] Denayer J. F. M.; De Vos D.; Leflaive P. Separation of Xylene Isomers. Met. Fram. Appl. From Catal. To Gas Storage 2011, 171–190. 10.1002/9783527635856.ch8.

[ref3] Tomás R. A. F.; Bordado J. C. M.; Gomes J. F. P. P-Xylene Oxidation to Terephthalic Acid: A Literature Review Oriented toward Process Optimization and Development. Chem. Rev. 2013, 113 (10), 7421–7469. 10.1021/cr300298j. 23767849

[ref4] Yang Y.; Bai P.; Guo X. Separation of Xylene Isomers: A Review of Recent Advances in Materials. Ind. Eng. Chem. Res. 2017, 56 (50), 14725–14753. 10.1021/acs.iecr.7b03127.

[ref5] Mazzotti M.; Storti G.; Morbidelli M. Optimal Operation of Simulated Moving Bed Units for Nonlinear Chromatographic Separations. J. Chromatogr. A 1997, 769 (1), 3–24. 10.1016/S0021-9673(97)00048-4.

[ref6] Ruthven D. M.; Ching C. B. Counter-Current and Simulated Counter-Current Adsorption Separation Processes. Chem. Eng. Sci 1989, 44 (5), 1011–1038. 10.1016/0009-2509(89)87002-2.

[ref7] Materials for Separation Technologies. Energy and Emission Reduction Opportunities. DOE, EERE Office: Oak Ridge, TN; 2005, 103. 10.2172/1218755.

[ref8] Koros W. J.; Lively R. P. Water and beyond: Expanding the Spectrum of Large-Scale Energy Efficient Separation Processes. AichE J. 2012, 58 (9), 2624–2633. 10.1002/aic.13888.

[ref9] Lively R. P.; Sholl D. S. From Water to Organics in Membrane Separations. Nat. Mater. 2017, 16 (3), 276–279. 10.1038/nmat4860. 28223707

[ref10] Koh D. Y.; McCool B. A.; Deckman H. W.; Lively R. P. Reverse Osmosis Molecular Differentiation of Organic Liquids Using Carbon Molecular Sieve Membranes. Science 2016, 353 (6301), 804–807. 10.1126/science.aaf1343. 27540170

[ref11] Banihashemi F.; Lin J. Y. S. B-Oriented MFI Zeolite Membranes for Xylene Isomer Separation - Effect of Xylene Activity on Separation Performance. J. Membr. Sci. 2022, 652, 120492 10.1016/j.memsci.2022.120492.

[ref12] Xomeritakis G.; Lai Z.; Tsapatsis M. Separation of Xylene Isomer Vapors with Oriented MFI Membranes Made by Seeded Growth. Ind. Eng. Chem. Res. 2001, 40 (2), 544–552. 10.1021/ie000613k.

[ref13] Sakai H.; Tomita T.; Takahashi T. P-Xylene Separation with MFI-Type Zeolite Membrane. Sep. Purif. Technol. 2001, 25 (1–3), 297–306. 10.1016/S1383-5866(01)00056-9.

[ref14] Yeong Y. F.; Abdullah A. Z.; Ahmad A. L.; Bhatia S. Separation of P-Xylene from Binary Xylene Mixture over Silicalite-1 Membrane: Experimental and Modeling Studies. Chem. Eng. Sci. 2011, 66 (5), 897–906. 10.1016/j.ces.2010.11.035.

[ref15] Wu X.; Wei W.; Jiang J.; Caro J.; Huang A. High-Flux High-Selectivity Metal-Organic Framework MIL-160 Membrane for Xylene Isomer Separation by Pervaporation. Angew. Chem., Int. Ed. 2018, 57 (47), 15354–15358. 10.1002/anie.201807935. 30248220

[ref16] Alemayehu H. G.; Hou J.; Qureshi A. A.; Yao Y.; Sun Z.; Yan M.; Wang C.; Liu L.; Tang Z.; Li L. Discrimination of Xylene Isomers by Precisely Tuning the Interlayer Spacing of Reduced Graphene Oxide Membrane. ACS Nano 2024, 18 (28), 18673–18682. 10.1021/acsnano.4c05461. 38951732

[ref17] Liu M.; Ge Y.; Du J.; Song Z.; Zhang C.; Zhou Q.; Zhang Y.; Gu X. Hierarchical MFI Zeolite Membranes for Superior Xylene Separation. Adv. Funct. Mater. 2024, 34, 2400772 10.1002/adfm.202400772.

[ref18] Ma Y.; Bruno N. C.; Zhang F.; Finn M. G.; Lively R. P. Zeolite-like Performance for Xylene Isomer Purification Using Polymer-Derived Carbon Membranes. Proc. Natl. Acad. Sci. U. S. A. 2021, 118 (37), e2022202118 10.1073/pnas.2022202118. 34493655 PMC8449391

[ref19] Ma Y.; Zhang F.; Deckman H. W.; Koros W. J.; Lively R. P. Flux Equations for Osmotically Moderated Sorption–diffusion Transport in Rigid Microporous Membranes. Ind. Eng. Chem. Res. 2020, 59 (12), 5412–5423. 10.1021/acs.iecr.9b05199.

[ref20] Jonquières A.; Clément R.; Lochon P.; Néel J.; Dresch M.; Chrétien B. Industrial State-of-the-Art of Pervaporation and Vapour Permeation in the Western Countries. J. Membr. Sci 2002, 206 (1–2), 87–117. 10.1016/S0376-7388(01)00768-2.

[ref21] UOP Parex^TM^ Process Handbook of petroleum refining processes; Meyers R. A. Ed.; McGraw-Hill Education, 2016.

[ref22] Peshev D.; Livingston A. G. OSN Designer, a Tool for Predicting Organic Solvent Nanofiltration Technology Performance Using Aspen One, MATLAB and CAPE OPEN. Chem. Eng. Sci. 2013, 104, 975–987. 10.1016/j.ces.2013.10.033.

[ref23] AmsterCHEM. Matlab CAPE-OPEN Unit Operation. https://www.amsterchem.com/matlabunitop.html. (accessed 2024 August 05).

[ref24] Wijmans J. G.; Baker R. W. The Solution-Diffusion Model: A Review. J. Membr. Sci. 1995, 107 (1–2), 1–21. 10.1016/0376-7388(95)00102-I.

[ref25] Cheng C.; Iyengar S. A.; Karnik R. Molecular Size-Dependent Subcontinuum Solvent Permeation and Ultrafast Nanofiltration across Nanoporous Graphene Membranes. Nat. Nanotechnol. 2021, 16 (9), 989–995. 10.1038/s41565-021-00933-0. 34239119

[ref26] Krishna R.; Baur R. Analytic Solution of the Maxwell–Stefan Equations for Multicomponent Permeation across a Zeolite Membrane. Chem. Eng. J. 2004, 97 (1), 37–45. 10.1016/S1385-8947(03)00149-9.

[ref27] Krishna R.; Wesselingh J. A. The Maxwell-Stefan Approach to Mass Transfer. Chem. Eng. Sci. 1997, 52 (6), 861–911. 10.1016/S0009-2509(96)00458-7.

[ref28] Ma Y.; Zhang F.; Yang S.; Lively R. P. Evidence for Entropic Diffusion Selection of Xylene Isomers in Carbon Molecular Sieve Membranes. J. Membr. Sci. 2018, 564, 404–414. 10.1016/j.memsci.2018.07.040.

[ref29] Bhattacharya N. Advances in Aromatics Technology: Lowers Paraxylene Cost of Production. Honeywell Technology Summit Kuwait: Kuwait; 2017, 3 4.

