# Supplementary material for: Techno-Economic Analysis of a Carbon Molecular Sieve-Based Xylene Isomer Purification Process
Source: Ind Eng Chem Res. 2024 Oct 11;63(42):18096–107. doi: 10.1021/acs.iecr.4c02180 (PMC11503610; doi:10.1021/acs.iecr.4c02180)
Supplement: Supplementary file 1 — ie4c02180_si_001.pdf [file ie4c02180_si_001.pdf]

Supporting information for

Techno-economic analysis of a carbon molecular sieve-based xylene isomer purification process

Conrad J. Roos [a,†], Hammed A. Balogun [a, †], Ryan P. Lively [a,*]

[a] School of Chemical and Biomolecular Engineering, Georgia Institute of Technology, Atlanta, GA 30332

[†] These authors made equal contributions.

*Corresponding Author: ryan.lively@chbe.gatech.edu

Table of Contents

Section S1. Three-stage OSRO cascade 3

Section S2. Three-stage pervaporation/OSRO hybrid cascade... 5

Section S3. Maxwell-Stefan transport flowchart for xylene separation 8

Section S4. Permeate purity for 1-stage OSRO/pervaporation xylene separation process............ 9

Section S5. Normalized energy requirement for 1-stage OSRO/pervaporation xylene separation process.. 10

Section S6. Equipment energy demand for 1-stage OSRO/pervaporation xylene separation process..11

Section S7. Membrane module area for 1-stage OSRO/pervaporation xylene separation process 13

Section S8. Cost distribution and sensitivity analysis for 1-stage OSRO/pervaporation separation process.. 14

Section S9. Permeate purity from three-stage membrane cascade systems................................. 16

Section S10. Permeance estimation 17

Section S1. Three-stage OSRO cascade

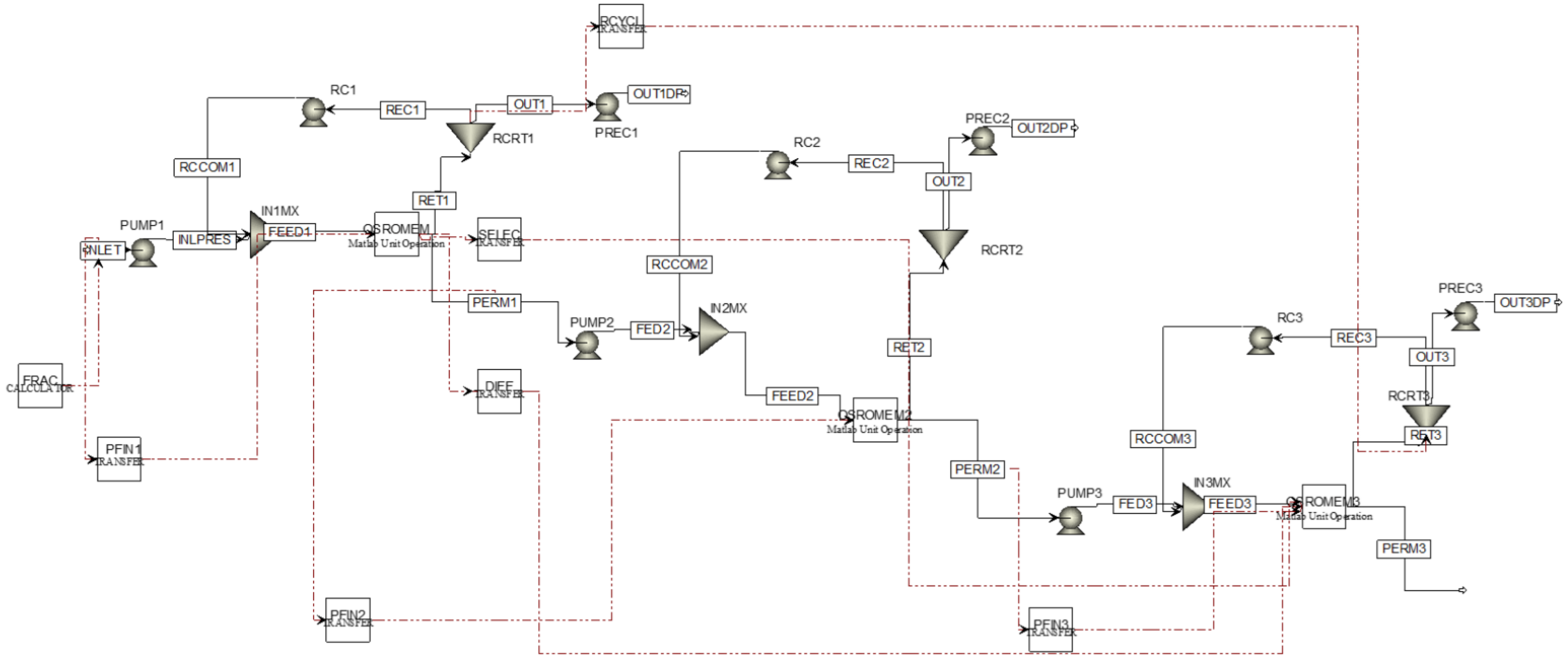

Figure S1. Aspen Plus process for a three-stage OSRO cascade process

Description of non-unit operation blocks

- Calculator FRAC: Used to control the inlet mole fraction. Uses a FORTRAN script to control inlet mole fraction by varying the flow rate of *p*-xylene.
- Transfer PFIN1, PFIN2, PFIN3: Loads the inlet flow rate of *p*-xylene into the CAPE-OPEN module. This allows for the calculation of the surface area necessary to achieve the desired recovery.
- Transfer DIFF: Ensures that all membrane modules are operating at the same *p*-xylene diffusivity.
- Transfer SELEC: Ensures that all membrane modules are operating at the same *p*-xylene/*o*-xylene selectivity.
- Transfer RCYCL: Ensures all splitters are using the same fraction for the streams exiting the system.

Unit operation identification

- Pumps: PUMP1, PUMP2, PUMP3, RC1, RC2, RC3
- Pressure Recovery Unit: PREC1, PREC2, PREC3
- Splitters/Mixers: IN1MX, IN2MX, IN3MX, RCRT1, RCRT2, RCRT3
- OSRO Unit: OSROMEM, OSROMEM2, OSROMEM3

Assumptions:

- Pump efficiency: 0.8.
- Pressure recovery unit efficiency: 0.9
- Split fraction in RCRT1, RCRT2, RCRT3 to OUT1, OUT2, OUT3: 0.05
- Feed pressure for OSRO units: 101 bar.

Section S2. Three-stage pervaporation/OSRO hybrid cascade

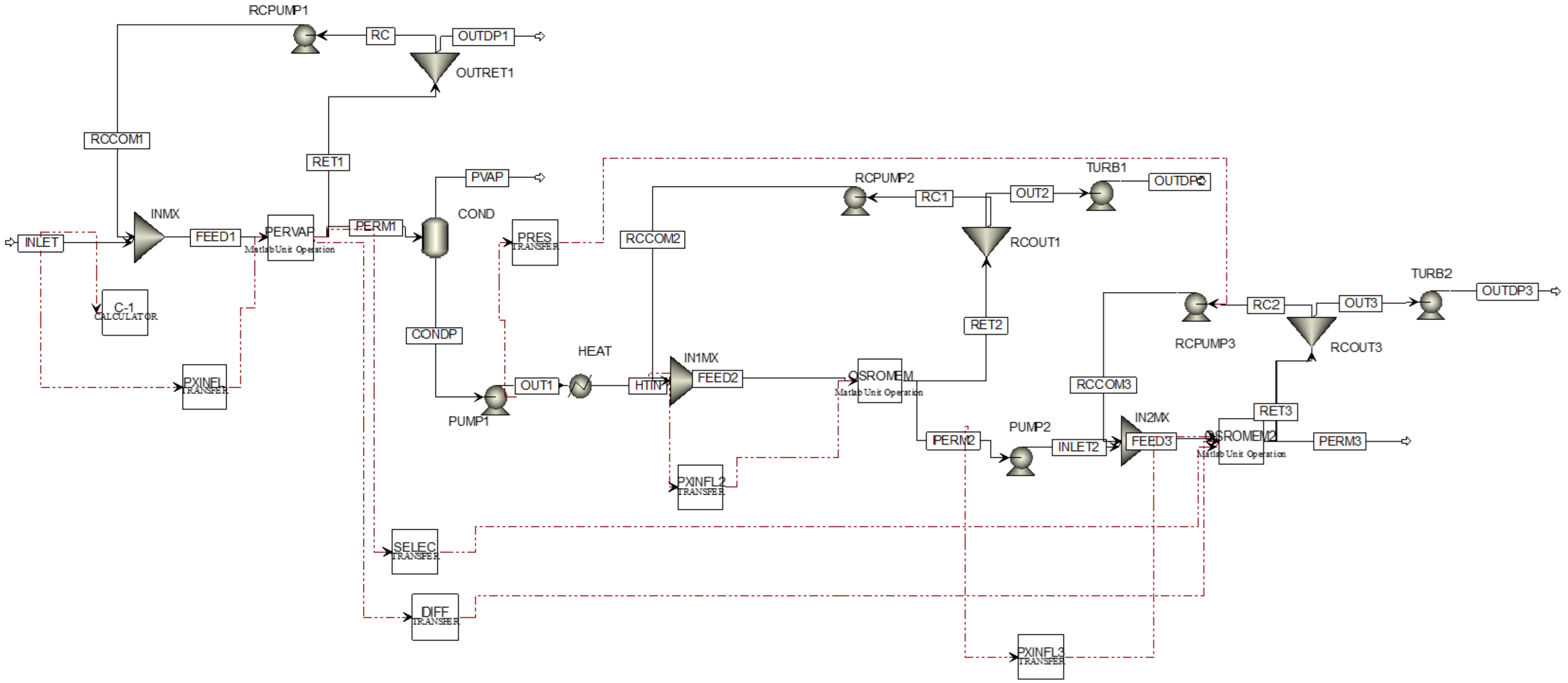

Figure S2. Aspen Plus process for a three-stage cascade, with an initial pervaporation stage followed by two OSRO stages.

Description of non-unit operation blocks

- Calculator C-1: Used to control the inlet mole fraction. Uses a FORTRAN script to control inlet mole fraction by varying the flow rate of *p*-xylene.
- Transfer PXINFL, PXINFL2, PXINFL3: Loads the inlet flow rate of *p*-xylene into the CAPE-OPEN module. This allows for the calculation of the surface area necessary to achieve the desired recovery.
- Transfer DIFF: Ensures that all membrane modules are operating at the same *p*-xylene diffusivity.
- Transfer SELEC: Ensures that all membrane modules are operating at the same *p*-xylene/*o*-xylene selectivity.
- Transfer RCYCL: Ensures all splitters are using the same fraction for the streams exiting the system.

Unit operation identification

- Pumps: PUMP1, PUMP2, RCPUMP1, RCPUMP2, RCPUMP3
- Pressure Recovery Units: TURB1, TURB2
- Splitters/Mixers: INMX, OUTRET1, IN1MX, RCOUT1, IN2MX, RCOUT3
- Heat Exchanger: HEAT
- Condenser: COND
- Pervaporaton Unit: PERVAP
- OSRO Unit: OSROMEM, OSROMEM2

Assumptions:

- Pump efficiency: 0.8
- Pressure recovery unit efficiency: 0.9

- Split fraction in OUTRET1, RCOUT1, RCOUTT3 to OUT1, OUT2, OUT3: 0.05
- Efficiency of heat recovery from condenser and heat exchanger: 0.8
- Heat exchanger (heater) efficiency: 0.9
- Feed pressure for OSRO units: 101 bar
- Permeate pressure for pervaporation: 0.5 kPa

Section S3. Maxwell-Stefan transport flowchart for xylene separation

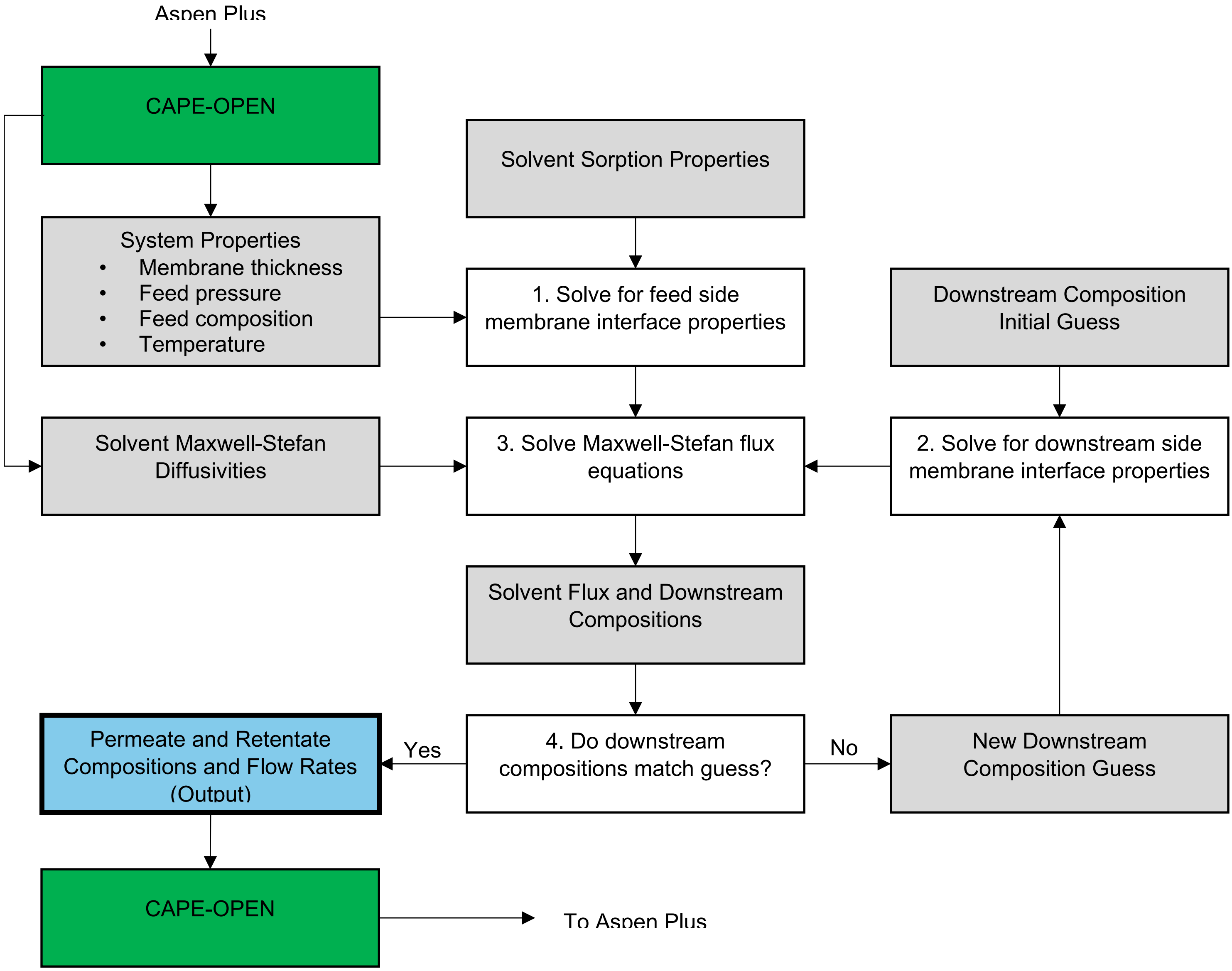

Figure S3. Flowchart of model for determination of OSRO performance for a carbon molecular sieve membrane. Feed parameters and membrane characteristics of interest are provided by Aspen Plus through the CAPE-OPEN interface, while the final retentate and permeate characteristics and membrane surface area are exported to Aspen Plus through CAPE-OPEN.

Section S4. Permeate purity for 1-stage OSRO/pervaporation xylene separation process

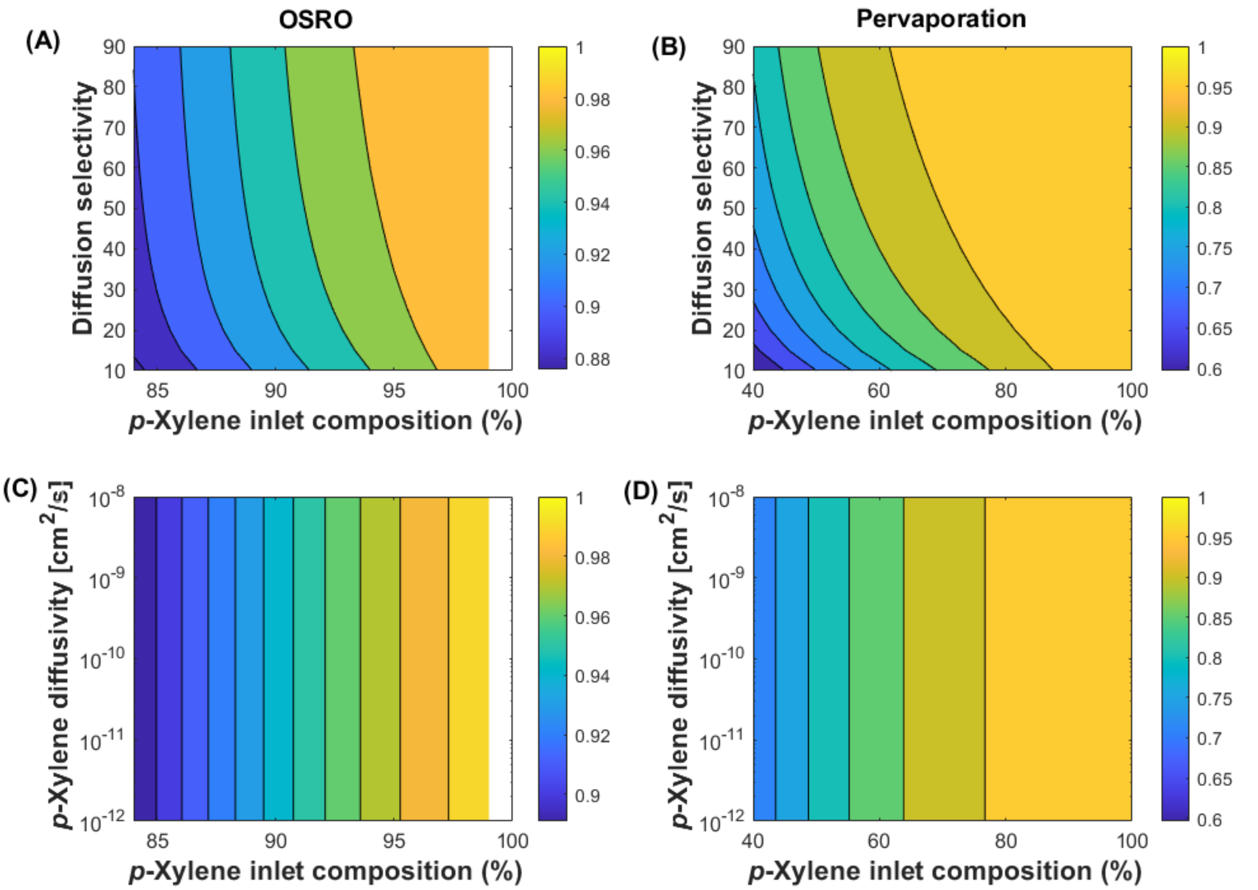

Figure S4. Permeate (p-xylene) purity for several combinations of inlet feed composition and (A& B) diffusion selectivity, and (C & D) p-xylene diffusivity. The left plot corresponds to the OSRO process, while the right is for the pervaporation module. P-Xylene recovery is fixed at 90%, and stage cuts were under 30%. Transmembrane pressure is 100 bar for OSRO and a vacuum pressure of 0.005 bar for pervaporation systems.

Section S5. Normalized energy requirement for 1-stage OSRO/pervaporation xylene separation process

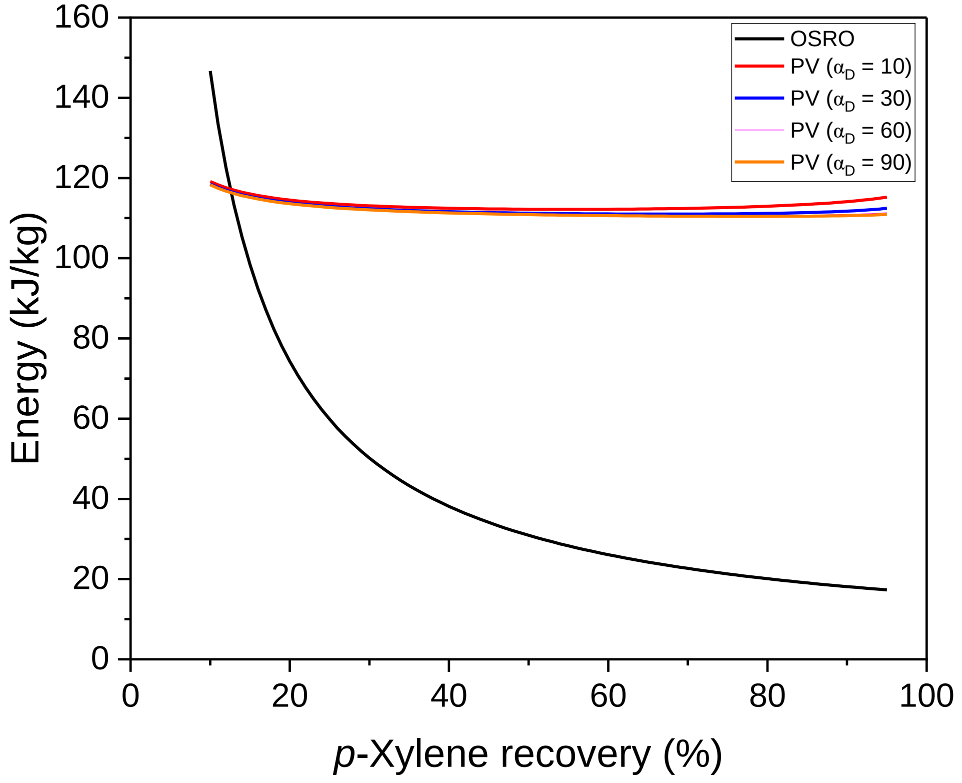

Figure S5. Comparison of energy required for xylene separation with increasing *p*-xylene recovery between 1-stage OSRO and pervaporation processes. Energy demand per kg of *p*-xylene product for OSRO does not vary with diffusion selectivity, while a slight variation is observed in the pervaporation process.

Section S6. Equipment energy demand for 1-stage OSRO/pervaporation xylene separation process

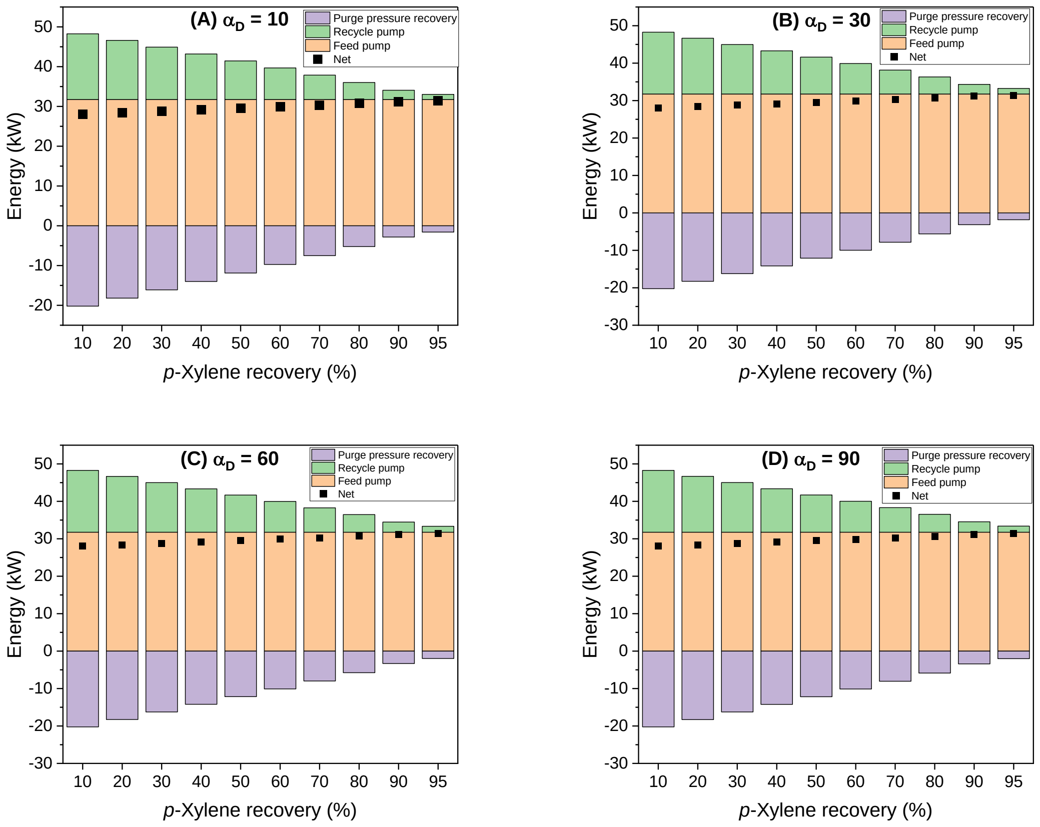

Figure S6. Energy requirement by equipment units in a 1-stage OSRO xylene separation at varying *p*-xylene recovery for different diffusion selectivity (α_D). OSRO energy demand is the net energy required for the feed pressurization, recycle stream pressurization plus the negative energy by the purge pressure recovery unit.

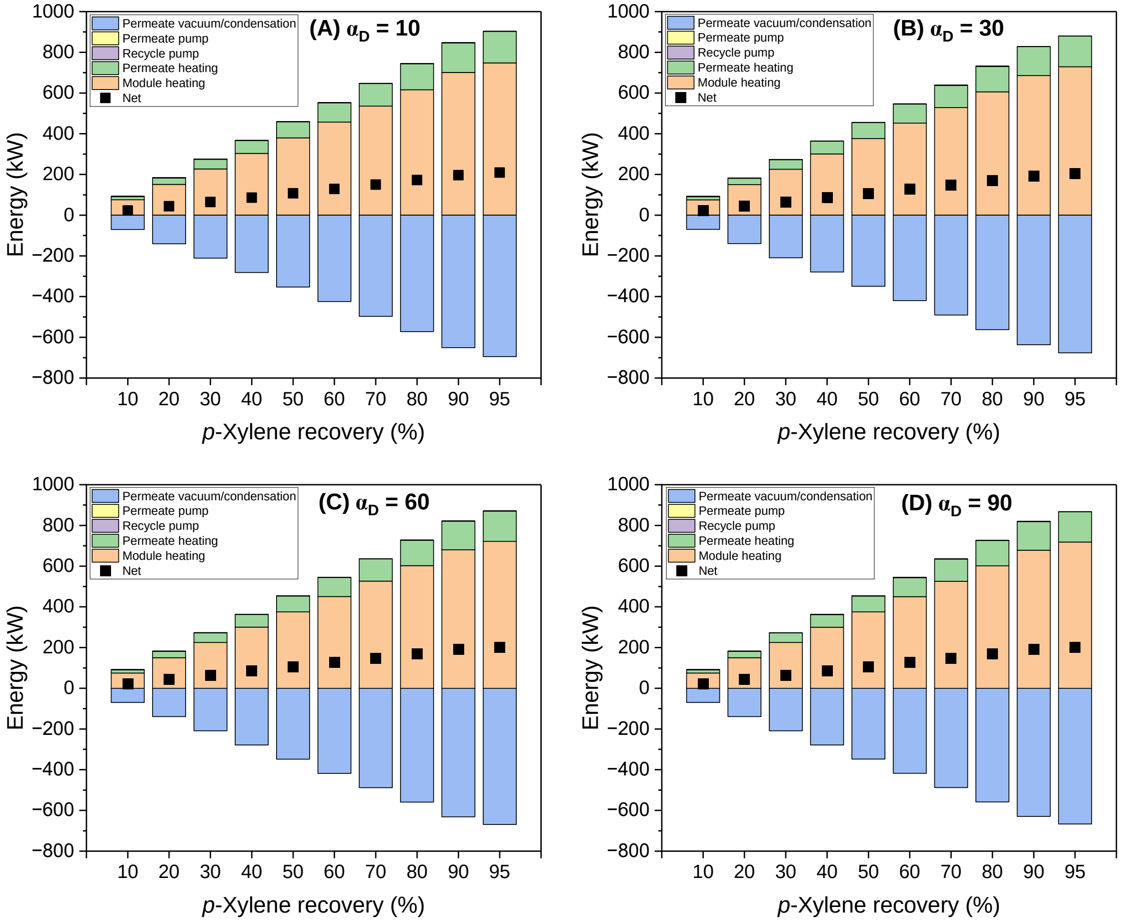

Figure S7. Energy requirement by equipment units in a 1-stage pervaporation xylene separation at varying *p*-xylene recovery for different diffusion selectivity (α_D). Pervaporation energy demand is the net energy required for the pervaporation module heating to prevent membrane freezing, recycle and permeate stream pressurization, heating of permeate stream plus the negative energy from the permeate vacuum/condensation unit. The black scatter plot represents the net energy demand in the process.

Section S7. Membrane module area for 1-stage OSRO/pervaporation xylene separation process

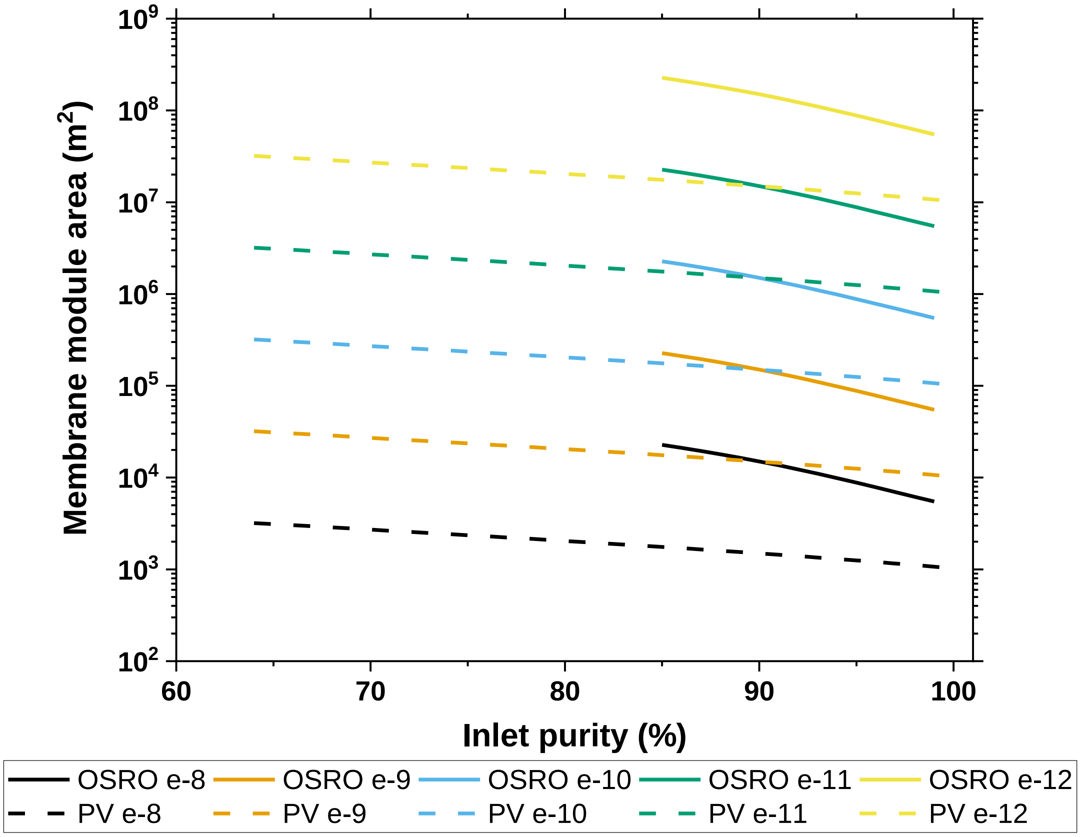

Figure S8. Required membrane areas for 1-stage OSRO/pervaporation xylene separation processes at varying *p*-xylene diffusivities (experimental $D_{p\text{-}Xy}$ of 10^{-10} cm^2/s). The diffusion selectivity of all membranes is 30; recovery is set at 90%, and all displayed data have *p*-xylene permeate purities exceeding 90%. Transmembrane pressure is 100 bar for OSRO and a vacuum pressure of 0.005 bar for pervaporation systems. A *p*-xylene diffusivity of 10^{-8} cm^2/s corresponds to a hydraulic permeance of 4.53 $Lm^{-2}h^{-1}bar^{-1}$ for the OSRO operating conditions, and a true permeance of 7.14 $cc(STP)cm^{-2}s^{-1}cmHg^{-1}$ for the pervaporation conditions (see Section S10).

Section S8. Cost distribution and sensitivity analysis for 1-stage OSRO/pervaporation separation process

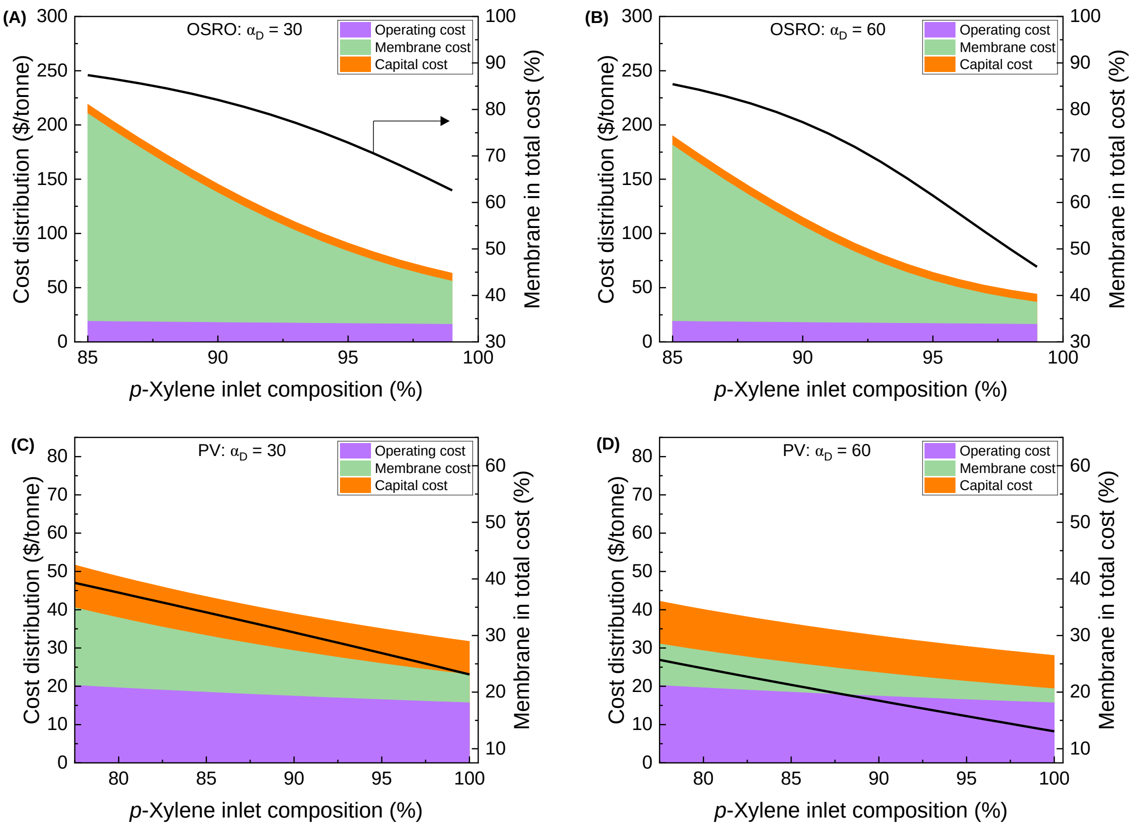

Figure S9. Analysis of cost components of single-stage OSRO (A & B) and pervaporation (C & D) processes for xylene separation at varying diffusion selectivities. Cost components are shown in a shaded area, while the percentage contribution of the membrane modules to total cost is represented with a black line (on the right). The operating and capital costs were obtained from the Aspen Process Economic Analyzer. All membranes had an *o*-xylene diffusivity of $8.74 \times 10^{-12}\ cm^2/s$; all displayed data have permeate *p*-xylene compositions >90%, and *p*-xylene recovery is set at 90%. Plots on the right for $\alpha_D = 30$ and $\alpha_D = 60$ on the left plots. Transmembrane pressure is 100 bar for OSRO and vacuum pressure of 0.005 bar for pervaporation systems with a feed temperature of 323K

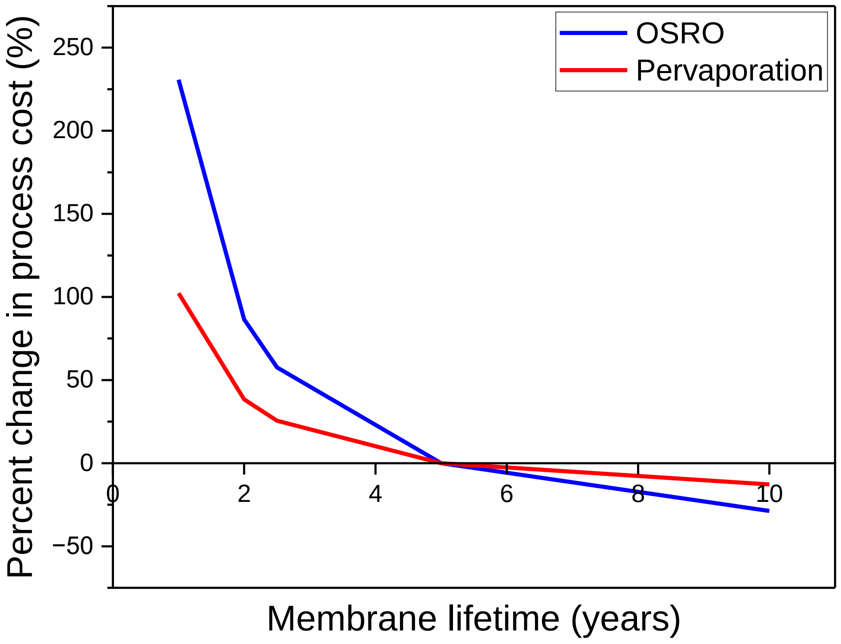

Figure S10. Sensitivity analysis of xylene separation process cost with change in membrane lifetime for an inlet feed mixture of 90% *p*-xylene, membrane diffusion selectivity of 30, and a target *p*-xylene recovery of 90%. The baseline membrane lifetime is 5 years.

Section S9. Permeate purity from three-stage membrane cascade systems

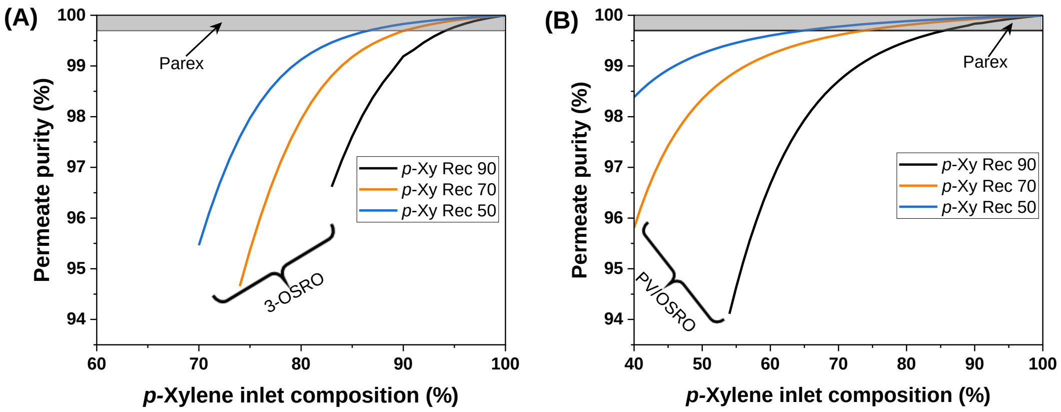

Figure S11. Permeate (*p*-xylene) purity from the last (third) membrane module for a three-stage OSRO cascade (3OSRO) and a pervaporation/two-stage OSRO hybrid (PV/2OSRO) across varied inlet composition. Number in the legend denotes the *p*-xylene recovery of the first module in each cascade. The shaded bank corresponds to the range of Parex purity (>0.997). Transmembrane pressure is 100 bar for OSRO and a vacuum pressure of 0.005 bar for pervaporation units.

Section S10. Permeance estimation

At a *p*-xylene diffusivity of 10^{-8} cm^2/s and inlet purity of 90%, the molar flux for the OSRO and pervaporation modules are $1.080 \times 10^{-4}\ \frac{mol}{cm^2 \cdot s}$ and $1.084 \times 10^{-3}\ \frac{mol}{cm^2 \cdot s}$ respectively. At these operating conditions, the *p*-xylene purity in the permeate for the two processes are 94.42% and 98.28% respectively.

Molar volumes for p-xylene and o-xylene are 123.3 and 120.6 cm^3/mol, respectively, while their vapor pressures at process temperature are 5.476 and 4.318 kPa, respectively. The activity coefficients of both species of xylene isomer are 1.

<u>Hydraulic permeance for the OSRO process</u>

The transmembrane pressure for the OSRO system is 100 bar (10,000 kPa)

The hydraulic permeance is calculated from flux as:

$$Permeance = \left(\frac{\mathbb{P}}{\ell}\right) = \frac{N^V}{\Delta P}$$

where $\left(\frac{\mathbb{P}}{\ell}\right)$ is permeance, N^V is the volumetric flux, and ΔP as transmembrane pressure

For *p*-xylene:

$$\left(\frac{\mathbb{P}}{\ell}\right)_{\text{p-Xy}} = \frac{(0.9442 \times 1.080 \times 10^{-4})\ mol}{cm^2 \cdot s}\ \frac{123.3\ cm^3}{mol}\ \frac{10^4\ cm^2}{m^2}\ \frac{1\ L}{10^3\ cm^3}\ \frac{3600\ s}{1\ h}\ \frac{1}{100\ bar}$$

$$\left(\frac{\mathbb{P}}{\ell}\right)_{\text{p-Xy}} = 4.53\ L \cdot m^{-2} \cdot h^{-1} \cdot bar^{-1}$$

For *o*-xylene:

$$\left(\frac{\mathbb{P}}{\ell}\right)_{\text{o-Xy}} = \frac{(0.0558 \times 1.080 \times 10^{-4})\ mol}{cm^2 \cdot s}\ \frac{120.6\ cm^3}{mol}\ \frac{10^4\ cm^2}{m^2}\ \frac{1\ L}{10^3\ cm^3}\ \frac{3600\ s}{1\ h}\ \frac{1}{100\ bar}$$

$$\left(\frac{\mathbb{P}}{\ell}\right)_{\text{p-Xy}} = 0.26\ L \cdot m^{-2} \cdot h^{-1} \cdot bar^{-1}$$

<u>True permeance for the pervaporation process</u>

For the pervaporation system, the upstream pressure is atmospheric (101 kPa) and the downstream with a vacuum pressure of 0.5 kPa.

The true permeance is calculated from flux as:

$$Permeance = \left(\frac{\mathbb{P}}{\ell}\right) = \frac{N^V}{\Delta f}$$

where $\left(\frac{\mathbb{P}}{\ell}\right)$ is permeance, N^V is the volumetric flux, and Δf as transmembrane fugacity. For the upstream side of the membrane, component fugacities are estimated as:

$$f_i^{up} = f_{i,0}^m = x_i^{up}\gamma_i^{up}P_i^{sat}\exp\left(\frac{\bar{V}_i}{RT}(P^{up} - P_i^{sat})\right)$$

$$f_{p-Xy}^{up} = 0.9 \times 1 \times 5.4757\ kPa \times \exp\left(\frac{123.3\ {}^{cm^3}/_{mol}}{8314\ \frac{cm^3 \cdot kPa}{mol \cdot K} \times 328.15\ K}(101 - 5.4757)\ kPa\right)$$

$$= 4.95\ kPa$$

$$f_{o-Xy}^{up} = 0.1 \times 1 \times 4.3182\ kPa \times \exp\left(\frac{120.6\ {}^{cm^3}/_{mol}}{8314\ \frac{cm^3 \cdot kPa}{mol \cdot K} \times 328.15\ K}(101 - 4.3182)\ kPa\right)$$

$$= 0.43\ kPa$$

For the downstream side, the component fugacities are estimated as:

$$f_i^{do} = f_{i,L}^m = x_i^{do}P^{do}$$

$$f_{p-Xy}^{do} = 0.9828 \times 0.5\ kPa = 0.491\ kPa$$

$$f_{o-Xy}^{do} = 0.0172 \times 0.5\ kPa = 0.009\ kPa$$

The transmembrane fugacities are obtained as:

$$\Delta f_{p-Xy} = (4.95 - 0.491)kPa = 4.459\ kPa$$

$$\Delta f_{o-Xy} = (0.43 - 0.009)kPa = 0.421\ kPa$$

The component fluxes in cm^3(STP)/cm^2.s are estimated as:

$$N_i^v\left(\frac{cm^3(STP)}{cm^2 \cdot s}\right) = N_i^v\left(\frac{cm^3|_{pervap}}{cm^2 \cdot s}\right) \times \frac{P^{do} \times 273.15K}{101.325kPa\ \times Temp}$$

$$= \left(N_i^m\left(\frac{mol|_{pervap}}{cm^2 \cdot s}\right) \times \left(\frac{R \times Temp}{P^{do}}\right)\right) \times \frac{P^{do} \times 273.15K}{101.325kPa\ \times Temp} = \left(N_i^m\left(\frac{mol|_{pervap}}{cm^2 \cdot s}\right) \times \left(\frac{R \times 273.15}{101.325}\right)\right)$$

$$N_{p-Xy}^v\left(\frac{cm^3(STP)}{cm^2 \cdot s}\right) = (0.9828 \times 1.084 \times 10^{-3})\left(\frac{mol|_{pervap}}{cm^2 \cdot s}\right) \times \left(\frac{8314 \times 273.15}{101.325}\right)$$

$$= 23.88 \ \frac{cm^3(STP)}{cm^2 \cdot s}$$

$$N_{o-Xy}^{v} \left(\frac{cm^3(STP)}{cm^2 \cdot s}\right) = (0.0172 \times 1.084 \times 10^{-3}) \left(\frac{mol|_{pervap}}{cm^2 \cdot s}\right) \times \left(\frac{8314 \times 273.15}{101.325}\right)$$

$$= 0.42 \ \frac{cm^3(STP)}{cm^2 \cdot s}$$

Thus, the true permeances are estimated as:

$$\left(\frac{\mathbb{P}}{\ell}\right)_{\mathrm{p-Xy}} = \frac{N_{p-Xy}^{v}}{\Delta f} = \frac{23.88}{4.459 \ \times 0.75} = 7.14 \ \frac{cm^3(STP)}{cm^2 \cdot s \cdot cmHg}$$

$$\left(\frac{\mathbb{P}}{\ell}\right)_{\mathrm{o-Xy}} = \frac{N_{o-Xy}^{v}}{\Delta f} = \frac{0.42}{0.421 \ \times 0.75} = 1.33 \ \frac{cm^3(STP)}{cm^2 \cdot s \cdot cmHg}$$
